# Mechanistic Pathways Underlying Genetic Predisposition to Atrial Fibrillation Are Associated With Different Cardiac Phenotypes and Cardioembolic Stroke Risk

**DOI:** 10.1161/CIRCGEN.124.004932

**Published:** 2025-06-17

**Authors:** Parag R. Gajendragadkar, Adam Von Ende, Federico Murgia, Alison Offer, C. Fielder Camm, Rohan S. Wijesurendra, Barbara Casadei, Jemma C. Hopewell

**Affiliations:** 1 Clinical Trial Service Unit and Epidemiological Studies Unit, Nuffield Department of Population Health (P.R.G., A.V.E., F.M., A.O., C.F.C., R.S.W., J.C.H.), University of Oxford, United Kingdom.; 2 Division of Cardiovascular Medicine, Radcliffe Department of Medicine (B.C.), University of Oxford, United Kingdom.

**Keywords:** atrial fibrillation, big data, genetics, ischemic stroke

## Abstract

**BACKGROUND:**

Genome-wide association studies have clustered candidate genes associated with atrial fibrillation (AF) into biological pathways reflecting different pathophysiological mechanisms. We investigated whether these pathways associate with distinct intermediate phenotypes and confer differing risks of cardioembolic stroke.

**METHODS:**

Three distinct subsets of AF-associated genetic variants, each representing a different mechanistic pathway, that is, the cardiac muscle function and integrity pathway (15 variants), the cardiac developmental pathway (25 variants), and the cardiac ion channels pathway (12 variants), were identified from previous AF genome-wide association studies. Using genetic epidemiological methods and large-scale datasets such as UK Biobank, deCODE, and GIGASTROKE, we investigated the associations of these pathways with AF-related cardiac intermediate phenotypes, which included electrocardiogram parameters (≈16 500 electrocardiograms), left atrial and ventricular size and function (≈36 000 cardiac magnetic resonance imaging scans), and relevant plasma biomarkers (N-terminal pro-B-type natriuretic peptide, ≈70 000 samples; high-sensitivity troponin I and T, ≈87 000 samples), as well as with subtypes of ischemic stroke (≈11 000 cases).

**RESULTS:**

Genetic variants representing distinct AF-related mechanistic pathways had significantly different effects on several AF-related phenotypes. In particular, the muscle pathway was associated with a longer PR interval (*P* for heterogeneity between pathways [*P*_het_]=1×10^−10^), lower left atrial emptying fraction (*P*_het_=5×10^−5^), and higher N-terminal pro-B-type natriuretic peptide (*P*_het_=2×10^−3^) per log-odds higher risk of AF compared with the developmental and ion-channel pathways. In contrast, the ion-channel pathway was associated with a lower risk of cardioembolic stroke (*P*_het_=0.04 in European, and 7×10^−^^3^ in multiancestry populations) compared with the other pathways.

**CONCLUSIONS:**

Genetic variants representing specific mechanistic pathways for AF are associated with distinct intermediate cardiac phenotypes and a different risk of cardioembolic stroke. These findings provide a better understanding of the etiological heterogeneity underlying the development of AF and its downstream impact on disease and may offer a route to more targeted treatment strategies.

Atrial fibrillation (AF) is the electrical end point of radically different pathophysiological pathways.^1,2^ Highlighting this complexity, linkage and family studies have associated AF with rare variants in genes encoding not just ion channels,^3,4^ but transcription factors,^5^ hormones,^6^ and more recently, cardiac structural proteins.^7–11^ In line with these findings, pathway and functional enrichment analyses from a large genome-wide association study (GWAS) have suggested that many of the genes annotated to AF-associated loci can be grouped into mechanistic pathways reflecting cardiac and skeletal muscle function and integrity, developmental events, hormone signaling, angiogenesis, and myocardial ion channels.^12^

In individuals with AF, many biomarkers and intermediate phenotypes have shown associations with subsequent AF-related outcomes, such as stroke.^13–15^ These findings may provide opportunities for improving the management and risk-stratification of patients with AF, as well as for repurposing drugs that are already available for treating other diseases.^12^ However, there remains uncertainty in our understanding of whether AF-associated mechanistic pathways are associated with distinct cardiac phenotypes and differing risks of severe complications.

Using clusters of genetic variants representing different AF-associated mechanistic pathways,^12^ we investigated associations between genetically predicted AF pathways and AF-related intermediate phenotypes, encompassing cardiac electrical, structural, and functional characteristics (ECG parameters,^16–18^ left atrial [LA] size and function,^19–21^ left ventricular [LV] function,^22,23^ NT-proBNP [N-terminal pro-B-type natriuretic peptide],^24,25^ and high-sensitivity cardiac troponins^25–27^), and examined their relevance to the risk of stroke.

## Methods

**Figure 1.**
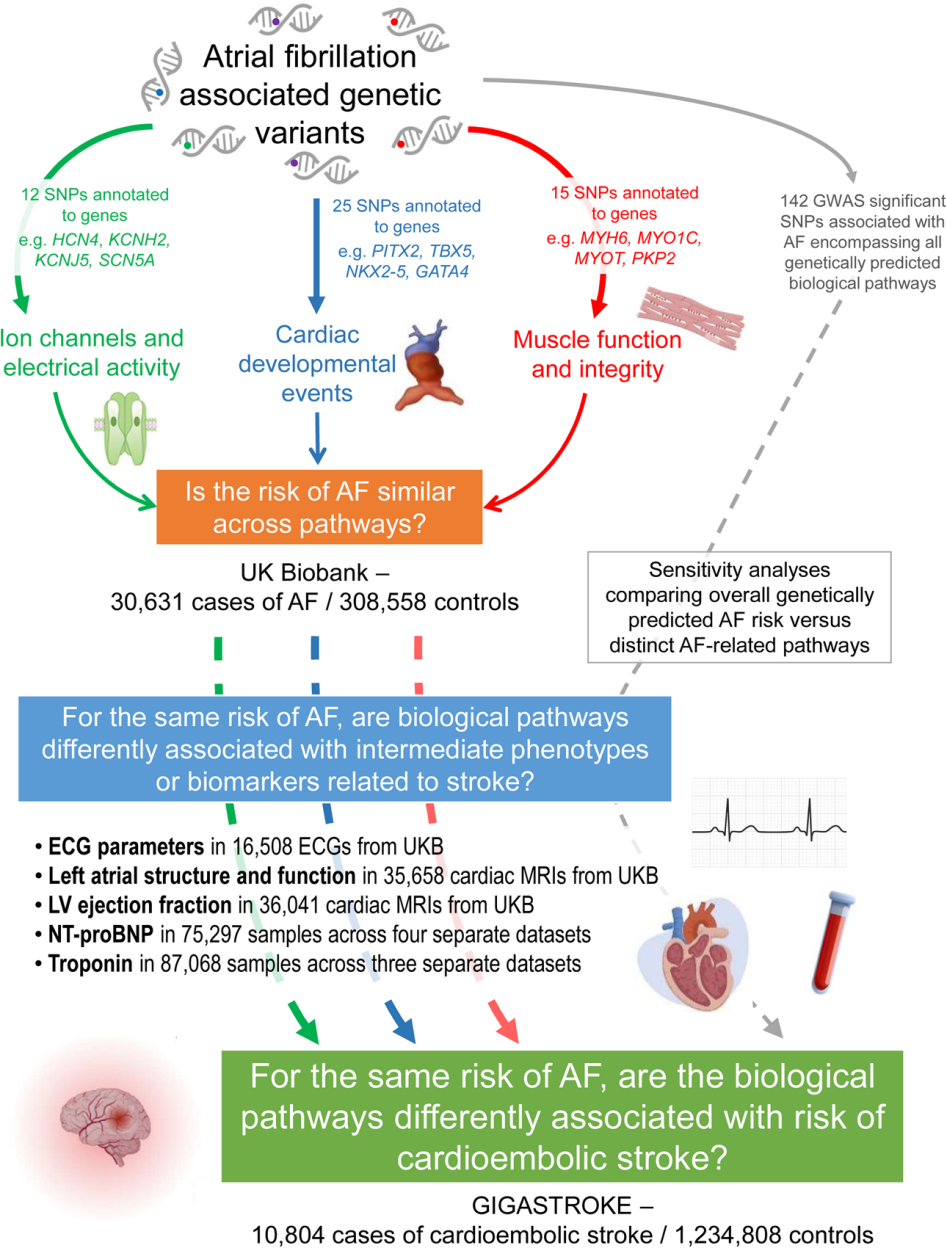
Summary of study design. AF indicates atrial fibrillation; GWAS, genome-wide association studies; LV, left ventricle; MRI, magnetic resonance imaging; N-terminal pro-B-type natriuretic peptide, NT-proBNP; and SNP, single nucleotide polymorphism.

A summary of the study design is shown in Figure 1, with detailed study methods presented in the Supplemental Methods and Tables S1 through S8.

### Data Availability

Data are available from the UK Biobank (https://biobank.ndph.ox.ac.uk/showcase/) in accordance with their published data access procedures. All other data are publicly available or were obtained from the corresponding author of the studies as detailed in the Supplemental Methods section and Table S5.

### Standard Protocol Approvals, Registrations, and Patient Consents

UK Biobank data were used from the UK Biobank Resource under Application Number 14568. All procedures and data collection in the UK Biobank were approved by the UK Biobank Research Ethics Committee (reference number 11/NW/0274), with participants providing full written informed consent for participation and subsequent use of their data for approved applications. Various publicly available nonidentifiable summary statistics were used in this study, with each of the multiple studies contributing to these datasets having consent procedures.

## Results

### Comparable Risks of AF Unrelated to Demographics or Comorbidities Across Mechanistic Pathways of Genetic Predisposition to AF

The estimated associations of AF of the 3 discrete pathways (muscle, 15 genetic variants; developmental, 25 variants; and ion channel, 12 variants) are based on the individual genetic variants’ associations in the AF GWAS meta-analysis (excluding the UK Biobank).^12^ As a validation step, we estimated the effects of these pathways on AF in the UK Biobank population. As anticipated, the pathways conferred comparable risks of AF (*P* for heterogeneity 0.72; Figure S1).

To investigate if the different pathways were associated with risk factors for AF, individual weighted genetic AF risk scores for each of the pathways were calculated in the UK Biobank. Participants were grouped into fifths based on each AF risk score, and characteristics in the top versus bottom fifth were compared (Table S9). As expected, other than the risk of AF, no meaningful differences in demographics, comorbidities, or baseline reported cardiovascular medication were noted.

### Differing Associations With Cardiac Intermediate Phenotypes Across Mechanistic Pathways for the Same Genetic Predisposition to AF

#### Surface ECG Parameters

**Figure 2.**
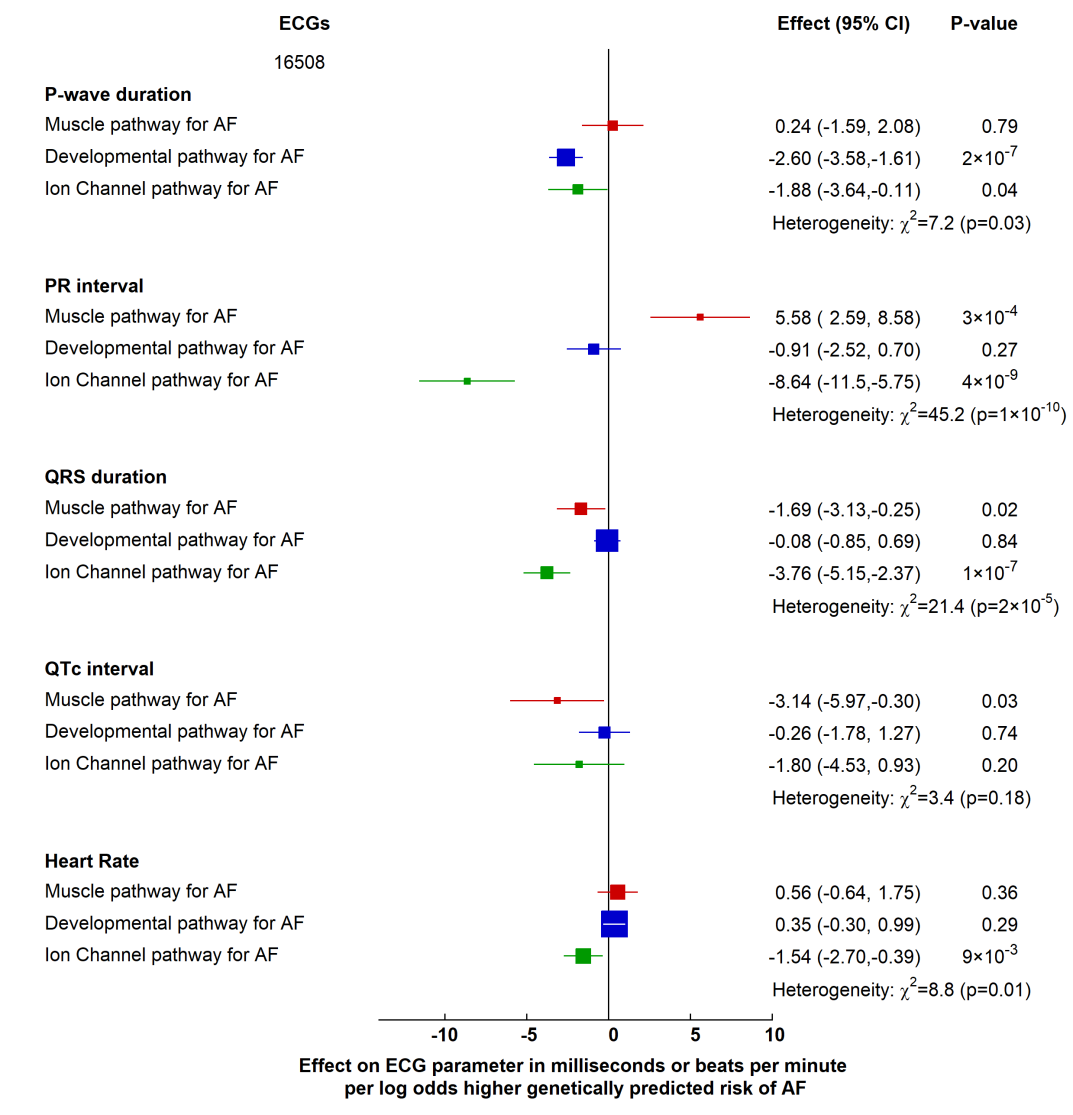
Effects of genetically predicted atrial fibrillation (AF) biological pathways on surface ECG parameters in the UK Biobank participants. Effect of genetically predicted AF biological pathways on ECG parameters (in milliseconds) or heart rate (in beats per minute) in a UK Biobank cohort of 16 508 participants without a history of AF. Boxes represent point estimates of effect per log-odds higher genetically predicted risk of AF, with their size inversely proportional to variance and solid lines representing 95% CIs. Effect sizes were calculated using inverse variance weighted methods, and heterogeneity was tested using the Cochran Q statistic.

In 16 508 participants from the UK Biobank with no history of AF, the genetically predicted AF pathways were associated with different effects on P-wave duration and PR interval (*P* for heterogeneity 0.03, and 1×10^−10^, respectively; Figure 2). In particular, the developmental and ion-channel pathways were associated with a shorter P-wave duration, but the muscle pathway was not (Figure 2). Conversely, the muscle pathway was associated with a longer PR interval (by 5.58 ms [95% CI, 2.59 to 8.58]; *P*=3×10^−4^ per log-odds higher genetically predicted risk of AF), while the ion-channel pathway was associated with a shorter PR interval (by 8.64 ms [95% CI, −11.53 to −5.75]; *P*=4×10^−^^9^ per log-odds higher genetically predicted risk of AF; Figure 2). This pattern remained the same after adjusting the ECG PR interval for the P-wave duration (*P* for heterogeneity 3×10^−^^10^; Figure S2).

There were small, but significant differences between the associations with QRS duration for the 3 pathways (*P* for heterogeneity 2×10^−^^5^; Figure 2), but not with corrected QT interval (*P* for heterogeneity 0.18; Figure 2). There were differences between the associations of the pathways with heart rate (*P* for heterogeneity 0.01; Figure 2), with a log-odds higher genetically predicted risk of AF via the ion-channel pathway being associated with a lower heart rate (−1.54 bpm [95% CI, −2.70 to −0.39]; *P*=9×10^−3^), whereas other pathways showed no association.

In contrast, overall AF genetic susceptibility (ie, as conferred by the 142-variant set) was not associated with differences in PR interval duration (*P*=0.33; Figure S3); however, was associated with a shorter P-wave (−1.25 ms [95% CI, −1.79 to −0.71]; *P=*6×10^−^^6^) and QRS duration (−0.95 ms [95% CI, −1.37 to −0.53]; *P*=1×10^−^^5^) per unit log-odds higher risk of AF.

These findings indicate that mechanistic pathways of genetic predisposition to AF have different associations with ECG parameters (most strikingly for the PR interval), which may not be apparent when overall genetic AF susceptibility is considered.

#### LA Size and Function

**Figure 3.**
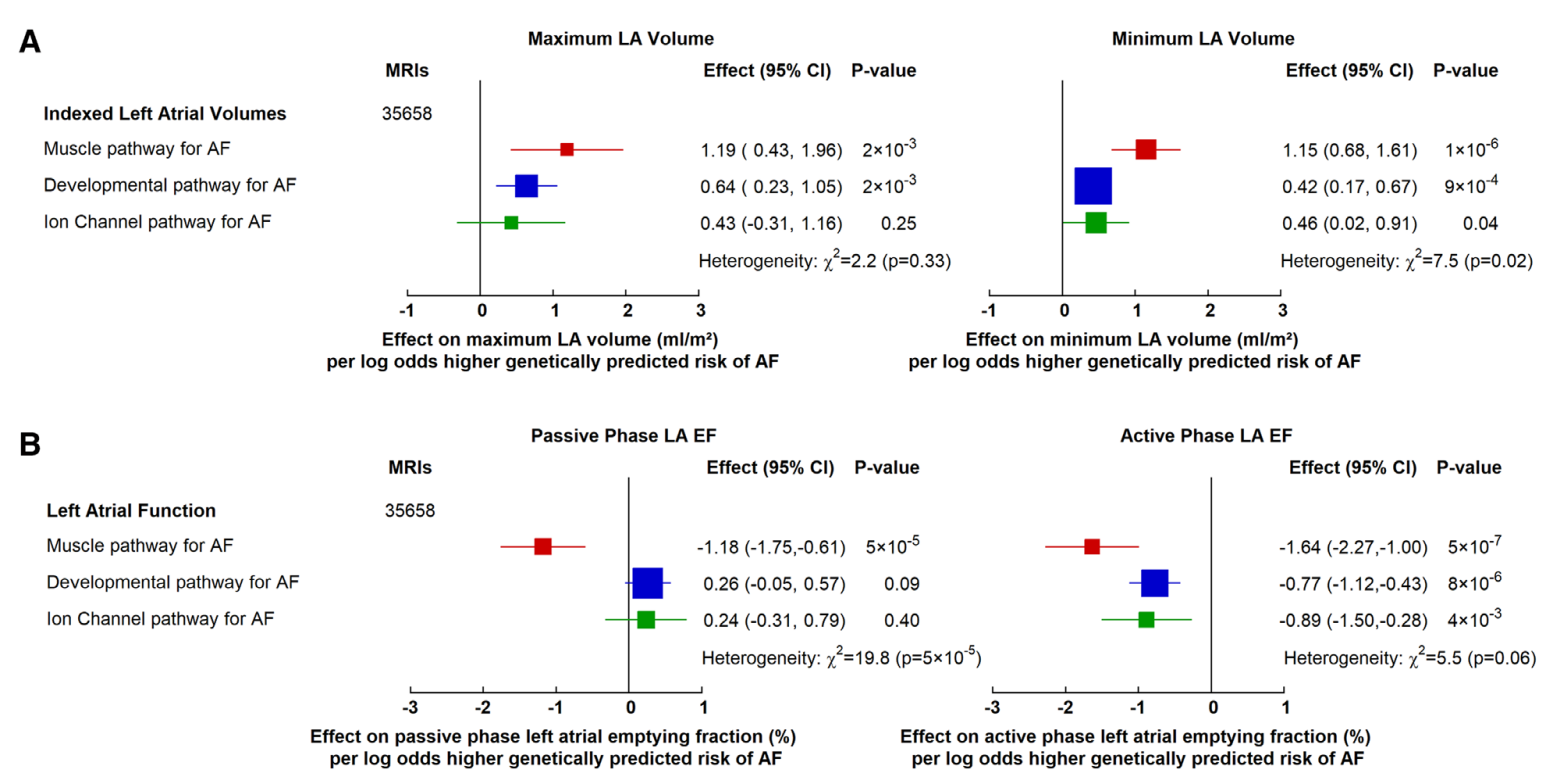
Effects of genetically predicted atrial fibrillation (AF) biological pathways on left atrial (LA) structure and function in the UK Biobank participants. Effect of AF pathways on (**A**) indexed LA volumes in mL/m^2^ and (**B**) LA passive and active phase emptying fractions from 35 658 cardiac magnetic resonance imaging (MRI) in the UK Biobank cohort. Boxes represent point estimates of effect per log-odds higher genetically predicted risk of AF, with their size inversely proportional to variance and solid lines representing 95% CIs. Effect sizes were calculated using inverse variance weighted methods from summary genome-wide association study data, and heterogeneity was tested using the Cochran Q statistic. EF indicates ejection fraction.

We then investigated the association of the pathways with LA structure and function using GWAS summary data from 35 658 UK Biobank participants who underwent a cardiac magnetic resonance imaging scan.^28^ While each AF pathway was similarly associated with larger maximum LA volumes (*P* for heterogeneity 0.33; Figure 3A), the magnitude of the associations with minimum LA volume differed across the pathways (*P* for heterogeneity 0.02; Figure 3A). Specifically, the muscle pathway was associated with a larger minimum LA volume compared with the developmental (*P*=7×10^−3^) and with the ion-channel pathway (*P*=0.04). The 142-variant set for overall AF susceptibility was associated with larger maximal and minimum LA volumes (Figure S4A).

When assessing LA function, we used previously defined passive and active phase LA emptying fraction measurements from cardiac magnetic resonance imaging.^28^ There were differences in the effects observed between different pathways for passive phase LA emptying fraction (*P* for heterogeneity 5×10^−^^5^). Specifically, a higher genetically predicted risk of AF via the muscle pathway was associated with a lower passive phase LA emptying fraction (−1.18% [95% CI, −1.75 to −0.61]; *P*=5×10^−^^5^); however, no association was observed with the other pathways (Figure 3B). The comprehensive 142-variant set for AF susceptibility was not associated with passive phase LA emptying fraction (Figure S4B).

In contrast, each of the 3 mechanistic pathways and the 142-variant set were associated with a lower active phase LA emptying fraction (Figure 3B; Figure S4B), and while the effect of the muscle pathway was the most extreme (as observed for the passive phase of LA emptying fraction), there was no significant difference between the pathways (*P* for heterogeneity 0.06; Figure 3B).

In summary, different mechanistic pathways of genetic predisposition to AF had different associations with LA structure and function. Among these, the muscle pathway was associated with larger LA size and more impaired LA function compared with the other pathways.

#### LV Function

LV dysfunction and AF share a complex bidirectional relationship. To investigate whether biologically informed pathways underpinning AF risk were differentially linked to imaging biomarkers of LV function, we examined their associations with LV ejection fraction using GWAS summary data from 36 041 UK Biobank participants who underwent a cardiac magnetic resonance imaging scan.^29^ None of the individual pathways were associated with LV ejection fraction, and there were no significant differences between pathways (Table S10). The muscle pathway was associated with a 0.65 mL/m^2^ lower indexed LV stroke volume (95% CI, −1.18 to −0.12; *P*=0.02), but associations did not differ significantly between the pathways (*P* for heterogeneity 0.14; Table S10). The 142-variant set was associated with a 0.17% lower LV ejection fraction (95% CI, −0.23 to −0.004; *P*=0.04) and a 0.20 mL/m^2^ lower indexed LV stroke volume (95% CI, −0.35 to −0.04; *P*=0.01).

#### NT-proBNP

We also examined associations with NT-proBNP using summary statistics from a cohort of 33 043 individuals from the UK Biobank Pharma Proteomics Project (UKB-PPP^30^; mean age, 59 years and similar to the whole UK Biobank cohort in terms of comorbidities) and a cohort of 35 559 Icelandic individuals from the deCODE+Icelandic Cancer Projects (ICP),^31^ mean age 55 years with a variety of comorbidities.

**Figure 4.**
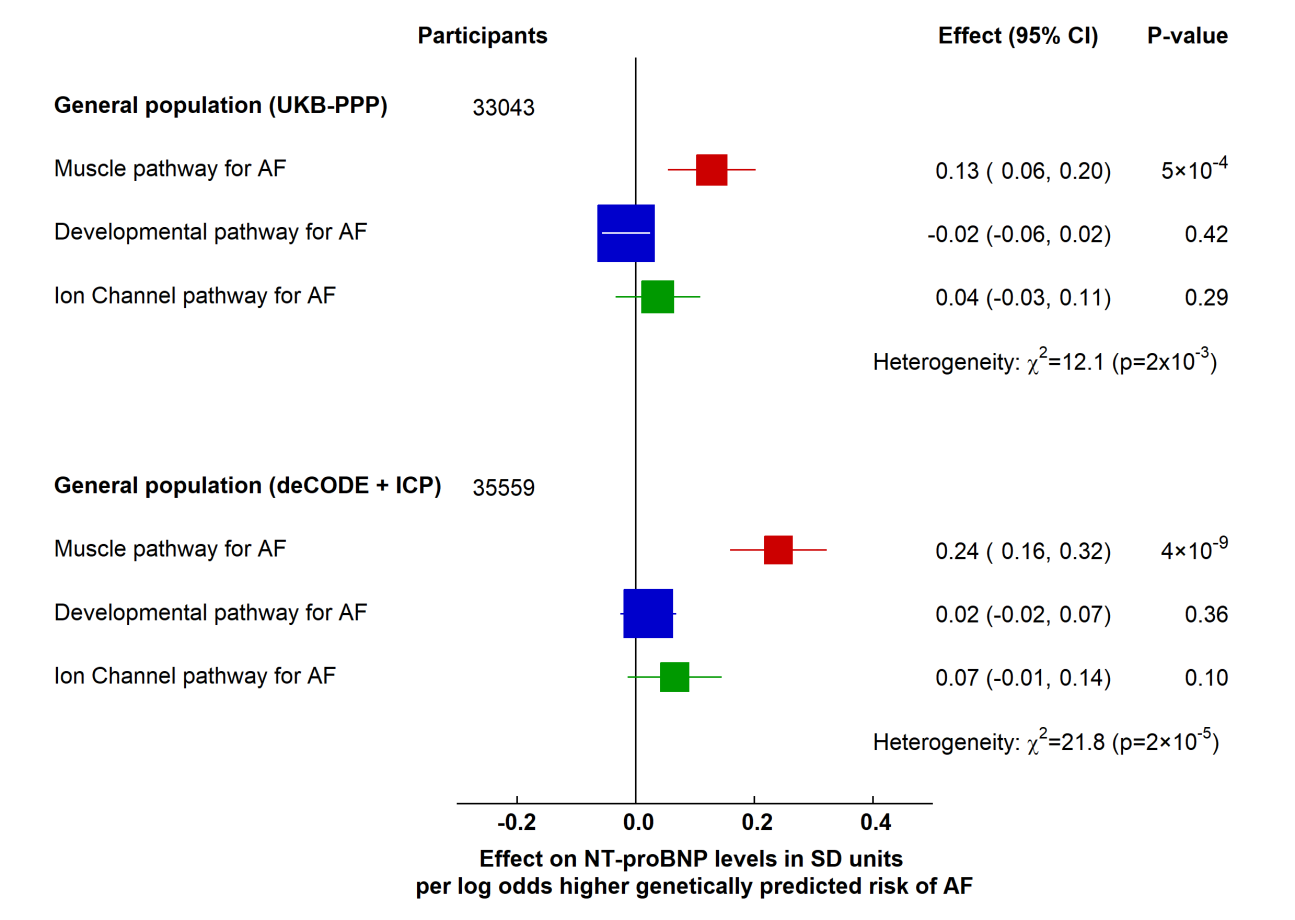
Effects of genetically predicted atrial fibrillation (AF) biological pathways on natriuretic peptide levels. Effect of AF pathways on N-terminal pro-B-type natriuretic peptide (NT-proBNP) in SD units in 33 043 British individuals from the UK Biobank Pharma Proteomics Project (UKB-PPP) and, in 35 559 Icelandic individuals from the deCODE and Icelandic Cancer Project (ICP). Boxes represent point estimates of effect per log-odds higher genetically predicted risk of AF, with their size inversely proportional to variance and solid lines representing 95% CIs. Effect sizes were calculated using inverse variance weighted methods from summary genome-wide association study data, and heterogeneity was tested using the Cochran Q statistic.

The biological pathways showed different associations with NT-proBNP (*P* for heterogeneity 2×10^−3^ and 2×10^−^^5^ for UKB-PPP and deCODE+ICP, respectively; Figure 4). In these 2 large cohorts, only the muscle pathway was associated with higher levels of NT-proBNP (0.13 SD unit higher [95% CI, 0.06–0.20]; *P*=5×10^−^^4^ in UKB-PPP and 0.24 SD unit higher [95% CI, 0.16 to 0.32]; *P*=4×10^−^^9^ in deCODE+ICP), with no associations noted for the other pathways (Figure 4).

To assess the sensitivity of these findings to population characteristics, we examined the associations in a population of 3301 healthy blood donors from the INTERVAL study^32^ (mean age, 43 years) and in 3394 older adults with risk factors for cardiovascular disease from the Intimal Medial thickening-PROgression as predictors of Vascular Events in a high-risk European population (IMPROVE) study^33^ (mean age, 65 years). Consistent with UKB-PPP and deCODE+ICP, only the muscle pathway was associated with a higher level of NT-proBNP, with heterogeneity between pathways seen in both cohorts (*P=*4×10^−3^ and *P*=0.04, respectively; Figure S5). The 142-variant set for overall risk of AF was also associated with higher levels of NT-proBNP, but only in the UKB-PPP and deCODE+ICP cohorts (Figure S6).

#### High-Sensitivity Troponins

To evaluate whether changes in LA function and biomarkers of LV dysfunction were linked to myocardial injury, we investigated associations with high-sensitivity troponin I (hsTnI; multiancestry population of 48 115 individuals^34^ and in a separate multiancestry population of 14 336 individuals free from prevalent coronary heart disease or heart failure^35^) and high-sensitivity troponin T (24 617 multiancestry individuals free from prevalent coronary heart disease or heart failure^35^) using GWAS summary data.

We found no differences between the biological pathways in terms of their associations with hsTnI or high-sensitivity troponin T (*P* for heterogeneity 0.14 and 0.36 for the hsTnI cohorts, and 0.85 for the high-sensitivity troponin T cohort; Figure S7).

The 142-variant set was not associated with higher hsTnI levels in the general population; however, it was associated with a slightly higher level of both troponin I and troponin T in multiethnic populations free from prevalent coronary heart disease or heart failure (Figure S8).

Overall, these data provide further evidence that different mechanisms underlying the genetic risk of AF are associated with distinct cardiac phenotypes and suggest that the muscle pathway is associated with biomarkers of LV dysfunction, and not myocardial injury, in addition to LA dysfunction.

### Genetic Predisposition to AF Conferred by Mechanistic Pathways Is Associated With Different Risks of Cardioembolic and Not Small-Vessel Ischemic Stroke

We next investigated whether different pathways of genetically predicted risk for AF may be associated with a different risk of cardioembolic stroke, for which AF is an important risk factor, and not with small-vessel ischemic stroke (as a negative control) because of its differing risk factor profile. We used summary statistics from the GIGASTROKE meta-analysis^36^ employing the Trial of ORG 10172 in Acute Stroke Treatment (TOAST) classification system^37^ to categorize acute stroke subtypes.

**Figure 5.**
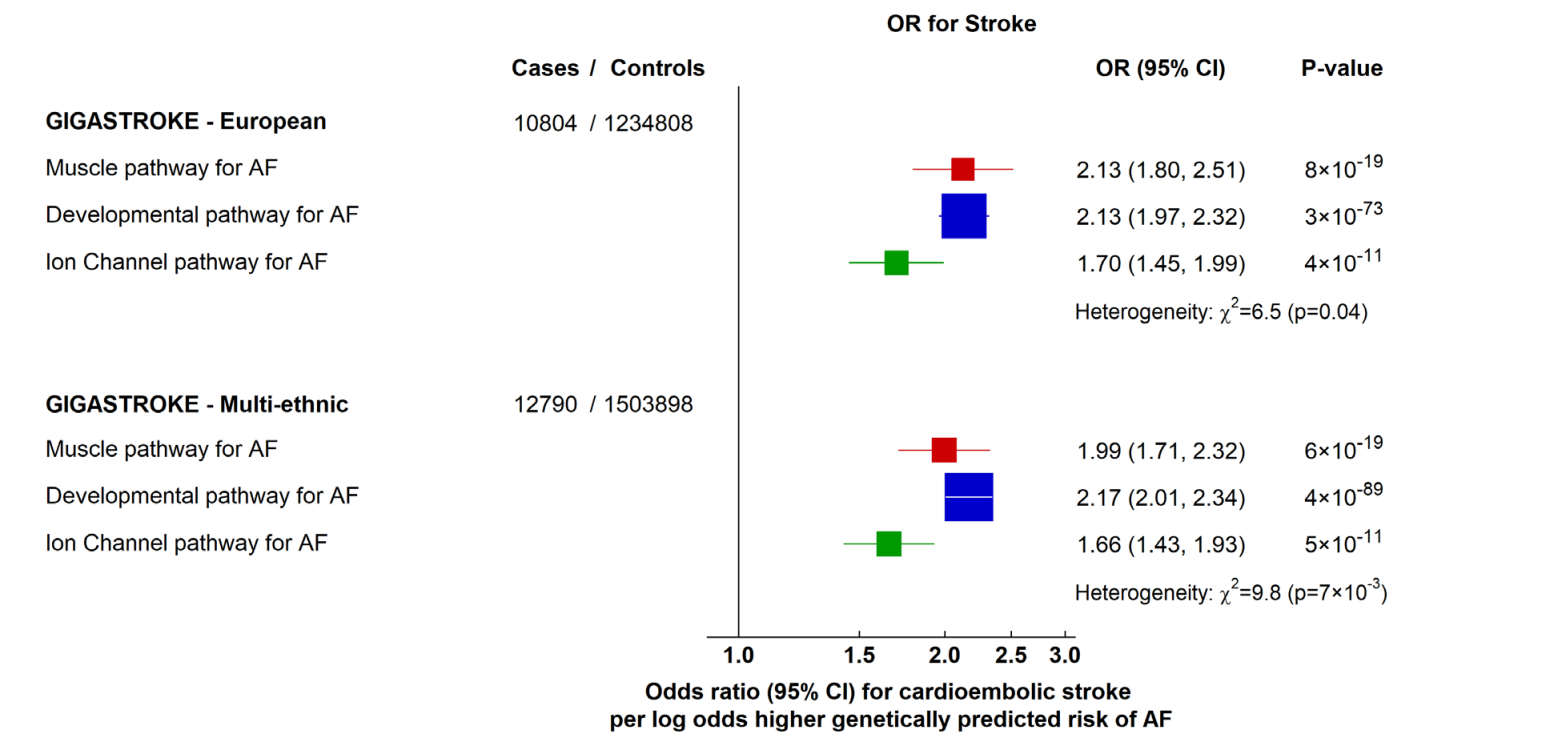
Effects of genetically predicted atrial fibrillation (AF) biological pathways on cardioembolic stroke. Odds ratio (OR) for developing cardioembolic stroke in the GIGASTROKE dataset. Boxes represent point estimates of effect per log-odds higher genetically predicted risk of AF, with their size inversely proportional to variance and solid lines representing 95% CIs. ORs were calculated using inverse variance weighted methods from summary genome-wide association study data, and heterogeneity was tested using the Cochran Q statistic.

In analyses limited to individuals of European ancestry (comparable with the population in which the AF GWAS was undertaken^12^), the 3 pathways were differentially associated with the risk of cardioembolic stroke (*P* for heterogeneity 0.04; Figure 5). Specifically, the ion-channel pathway was associated with a lower risk of cardioembolic stroke, most profoundly when compared with the developmental pathway (*P*=0.01). In the larger multiancestry GIGASTROKE meta-analysis, we were similarly able to replicate the differences in the risk of cardioembolic stroke across the different pathways (*P* for heterogeneity 7×10^−3^), again with a lower risk conferred by the ion-channel pathway (odds ratio for cardioembolic stroke, 1.66 [95% CI, 1.43 to 1.93] per log-odds higher genetically predicted risk of AF), particularly when compared with the developmental pathway (*P*=1×10^−3^; Figure 5). By contrast, none of the individual pathways was associated with small-vessel ischemic stroke, with no significant heterogeneity among the estimates (*P* for heterogeneity 0.20; Figure S9). Consistent with previous Mendelian randomization studies of AF and stroke,^38,39^ the 142-variant set for overall AF susceptibility was associated with a higher risk of cardioembolic stroke, and not with small-vessel ischemic stroke (Figure S10).

Overall, these findings suggest that different mechanisms underlying the genetic risk of AF are not only associated with distinct intermediate phenotypes, but also with different risks of cardioembolic stroke.

### Sensitivity Analyses

#### Investigations for Pleiotropic Effects Within the Pathways

Sensitivity analyses conducted for each phenotype did not suggest pleiotropy and indicated that effect estimates were robust to methodological assumptions. Further details are available in the Supplemental Results section and Table S11. Funnel plots to visualize the effects of each variant on risk of cardioembolic stroke within each AF score are shown in Figures S11 through S13, and leave-one-out analyses suggested results were robust to the exclusion of individual genetic variants (Figures S14 through S16).

#### Potential Effects of Pathway-Associated Intermediate Phenotypes on Risk of Cardioembolic Stroke

We performed analyses for cardioembolic stroke conditional on the effects of the variants on the intermediate phenotypes, grouped by ECG parameters, markers of LA size and function, and then NT-proBNP and LV ejection fraction. Estimates were comparable with the primary analyses even after accounting for these effects (Tables S12 through S14), suggesting that associations between the variants and intermediate phenotypes examined in this study are unlikely to fully explain the differences in association with cardioembolic stroke.

## Discussion

Our findings indicate that distinct biological pathways underlying the genetically predicted risk of AF are differentially associated with the atrial and ventricular phenotypes, and, importantly, with the risk of cardioembolic stroke. This study not only validates the heterogeneous nature of the disease processes predisposing to AF, but highlights the potential for improved risk-stratification and more targeted therapeutic strategies.

### Biological Pathways for AF

A variety of biological pathways underpinning the development of AF have been identified using clinical, laboratory, and animal methods.^1,2^ Commensurate with early understanding of AF as a primarily electrical disease, initial linkage analyses identified ion-channel genes as potentially relevant in the pathogenesis of AF.^3,40^ More recently, large GWAS have identified many common genetic variants underlying AF susceptibility and have used an array of in silico approaches to suggest their relevance to distinct biological pathways.^12^ Within this framework, however, combining information across the genome without biological considerations could obscure important mechanistic distinctions, prompting us to investigate the relevance of discrete biologically informed genetic pathways associated with AF risk.

Our findings suggest that different AF-related mechanisms have different effects on biomarkers, intermediate phenotypes, and risk of cardioembolic stroke, which may partially explain the heterogeneity seen in observational studies linking ECG,^16–18^ cardiac imaging parameters,^19–22^ and natriuretic peptides^41,42^ with risk of AF.

### Atrial and Ventricular Substrates

Changes in atrial and ventricular substrates in the context of AF are complex and have been well described,^1,43^ with several intermediate phenotypes affecting atria and ventricles being relevant to AF (eg, P-wave duration or QT interval,^16,17^ LA volume^44^ or emptying fraction,^45^ and LV dysfunction^22^). We initially investigated whether genetic variation associated with risk of AF and representing 3 distinct pathways (ion channel, muscle structure and function, and developmental pathways) had different effects on the intermediate phenotypes in individuals in sinus rhythm.

Out of the surface ECG parameters, most strikingly, the muscle and the ion-channel pathways had directionally opposite associations with the PR interval. Strong associations between the pathways with P-wave duration were not seen in our study, and thus the heterogeneity seen for associations with the PR interval persisted even after adjusting for P-wave duration. This may suggest that variations in AV node transit time, rather than measures of atrial depolarization, reflect different biological pathways to AF and related outcomes. However, ECG parameters in the UK Biobank are based on automated ECG recordings and measuring P-waves using automated ECG algorithms has considerable challenges given their low amplitude and variability across different ECG leads,^46,47^ so it is possible that the PR interval associations simply more accurately reflect atrial electrical parameters or include atrial repolarization which the P-wave duration may not.

Observational data have suggested that both short and long P-wave durations and PR intervals are associated with a higher risk of AF,^17,18^ and Mendelian randomization studies have suggested shorter atrial ECG parameters are associated with a higher risk of AF, commensurate with the effects of the ion-channel pathway.^48^ Our findings are consistent with data linking an atrial cardiomyopathic substrate to slower atrial conduction,^49–51^ whereas shorter PR intervals may reflect shortening of atrial action potential duration (as seen in those with AF^52^) and more rapid conduction. Further investigation would be needed to establish whether these insights also provide information on the mechanisms underpinning AF development (eg, reentrant versus multiple wavelets, or trigger-dependent versus substrate-dependent) and potentially therapy (eg, response to anti-arrhythmic drugs or catheter ablation).

Our study showed differing associations of the AF-associated biological pathways with NT-proBNP, with the muscle pathway being associated with higher plasma levels across all cohorts, including in seemingly healthy, younger subjects. We also observed associations between the muscle and ion-channel pathways and QRS interval duration on ECG, and that the muscle pathway was associated with a lower indexed LV stroke volume. The muscle pathway also had the strongest associations with lower LA function, particularly in the passive emptying phase, which has been suggested to be an early marker of LV diastolic dysfunction.^53^

Associations between NT-proBNP and AF have been reported widely in the literature alongside a consistently increased risk of stroke.^24,25^ Our previous work also indicated that people with lone AF continue to show evidence of subtle ventricular dysfunction and impaired myocardial energetics on cardiac magnetic resonance imaging/spectroscopy, despite normalization of rhythm ≈6 months postablation, consistent with the presence of a subclinical cardiomyopathy underpinning AF.^22^

Our analyses did not support any consistent difference in the effect of AF-associated pathways on hsTnI/T levels in a general population, or separately in a cohort of individuals free from coronary artery disease or heart failure. In subjects with AF, hsTnI/T levels are relevant to risk-stratification for future cardiovascular outcomes.^25–27^ Although the muscle pathway in particular was associated with markers of LA and LV dysfunction, this does not necessarily reflect the presence of myocardial injury and cell death. Other findings suggest that this relationship may be accounted for, at least in part, by concomitant coronary disease.^54^

### Different Risks of Stroke Based on Different Pathways to AF

Our study suggests that genetic variants representing different pathways associated with the risk of AF can confer differing risks of cardioembolic stroke. There is evidence suggesting that structural or electrical changes within the atria may lead to thromboembolism and AF independently.^44,55–57^ Furthermore, missense mutations in genes associated with changes in atrial structure confer an increased risk of both AF and stroke.^8,9,58^ This has given rise to the hypothesis that AF and stroke may be the distinct end points of a cardiomyopathic substrate.^15,59–62^ In our study, the same risk of AF underpinned by different biological pathways was associated with differences in the risk of cardioembolic stroke.

We demonstrated associations between the ion-channel pathway and ECG parameters, as well as the muscle pathway and some of the known intermediate phenotypes for atrial and ventricular cardiomyopathy, however the developmental pathway for AF was associated with a higher risk of stroke in the absence of an obvious atrial phenotype as assessed by conventional biomarkers. Although the function of the specific variants included in the developmental pathway (*PITX2*, *NKX2-5*, *GATA 4*, and *TBX5*) is only partially known,^4^ these genes may impact cardiac development in early life, with evidence that missense mutations in *TBX5* can lead to profound atrial transcriptional alteration and AF.^63^ Investigation of more sophisticated and novel biomarkers (eg, bone morphogenetic protein 10^64,65^) may yield more insights into understanding how this pathway impacts AF and stroke risk.

Finally, in addition to illustrating the heterogeneity between the effects of different AF pathways on stroke risk, we have been able to exploit the strengths of the Mendelian randomization methodologies used (and extensive sensitivity analyses to ensure robustness) to provide support for a causal role of each of the AF pathways examined for risk of stroke. Further studies are needed to understand if individuals with AF who have risk profile patterns associated with different biological mechanisms may derive more benefit from specific interventions and help inform treatment pathways (eg, for stroke prevention or development of heart failure, and response to antiarrhythmic drugs or catheter ablation).

### Limitations

To define our biological pathways, we used the largest GWAS for AF available at the time of our study and its multimodal in silico gene and pathway annotations, and selected variants meeting GWAS significance.^12^ Pathway annotation is complex and based on existing knowledge of the functions of genes, and thus limited by new scientific discovery. Although the associations observed with different intermediate phenotypes validate the different pathways represented to some extent, we did not comprehensively test distinct biological pathways as a whole, but rather specific variants within these pathways known to be associated with AF. Further identification of specific rare variants and consequent biological investigation would refine these insights.

The GWAS for AF used for genetic variant selection was conducted in participants of European ancestry, and the various outcomes considered were also chiefly examined in those of European ancestry, therefore further studies are needed to assess the generalizability of our findings to more diverse populations.

### Conclusions

Genetic variants representing distinct biological pathways for AF susceptibility have differing effects on intermediate cardiac electrical, structural, and functional phenotypes, extending to the ventricle, and risk of cardioembolic stroke. These findings provide a better understanding of the etiological heterogeneity driving the development of AF and may offer a route to genetically informed treatment strategies.

## ARTICLE INFORMATION

### Sources of Funding

This work was supported by the British Heart Foundation (BHF; grant numbers FS/17/17/32438 [P.R. Gajendragadkar and B. Casadei], FS/20/15/34920 [C.F. Camm and J.C. Hopewell], RG/16/12/3245 and CH/12/3/29609 [B. Casadei], and FS/14/55/30806 and CH/1996001/9454 [J.C. Hopewell]). The authors also acknowledge support from the European Union (grant agreement 633196-CATCH ME); BHF Oxford Centre of Research Excellence, National Institute for Health and Care Research (NIHR) Oxford Biomedical Research Centre, and the Nuffield Department of Population Health, University of Oxford, United Kingdom. The funders had no role in the study design, data collection or analysis, preparation of the article, or decision to publish.

### Disclosures

P.R. Gajendragadkar, A.V. Ende, F. Murgia, A. Offer, C.F. Camm, R.S. Wijesurendra, and J.C. Hopewell work at the Clinical Trial Service Unit and Epidemiological Studies Unit, Nuffield Department of Population Health, which receives research grants from industry that are governed by University of Oxford contracts that protect its independence, and has a staff policy of not taking personal payments from industry; further details can be found at https://www.ndph.ox.ac.uk/about/independence-of-research. B. Casadei is supported by a BHF personal chair; her research is funded by BHF programme grants, the BHF Oxford Centre for Research Excellence and the NIHR Oxford Biomedical Research Centre. She also receives in-kind research support from iRhythm and Roche Diagnostics. J.C. Hopewell and her research is supported by the BHF, NIHR Oxford Biomedical Research Centre, the Nuffield Department of Population Health, the BHF Oxford Centre for Research Excellence, and by grants from industry held in accordance with the policy detailed above.

### Supplemental Material

Supplemental Methods

Supplemental Results

Tables S1–S14

Figures S1–S16

References 66–73

## Supplementary Material
