# Supplementary material for: Mechanistic Pathways Underlying Genetic Predisposition to Atrial Fibrillation Are Associated With Different Cardiac Phenotypes and Cardioembolic Stroke Risk
Source: Circ Genom Precis Med. 2025 Jun 17;18(3):e004932. doi: 10.1161/CIRCGEN.124.004932 (PMC12173164; doi:10.1161/CIRCGEN.124.004932)
Supplement: Supplementary file 1 [file hcg-18-e004932-s001.pdf]

SUPPLEMENTAL MATERIAL

Supplemental Methods…. 1
Genetically-predicted mechanistic pathways associated with risk of AF 1
Genetic Instruments used in study 1
Study populations and outcome definitions 3
Atrial fibrillation and demographics 3
ECG parameters 3
Cardiac MRI parameters 3
NT-proBNP 3
High-sensitivity troponin I and T (hsTnI, hsTnT) 4
Ischaemic stroke 4
Statistical analysis 4
Primary analyses 4
Sensitivity analyses 4
Supplemental Results……. 6
Supplemental Table S1. Genetic variants included in 142-variant set for comprehensive AF risk 7
Supplemental Table S2. Genetic variants included in muscle pathway for AF 11
Supplemental Table S3. Genetic variants included in developmental pathway for AF 12
Supplemental Table S4. Genetic variants included in ion channel pathway for AF 13
Supplemental Table S5. Details of datasets used in study 14
Supplemental Table S6. Phenotype definition of Atrial Fibrillation in UK Biobank 16
Supplemental Table S7. Phenotype definitions of other diseases in UK Biobank 17
Supplemental Table S8. Code definitions for medications 18
Supplemental Table S9. Characteristics of top and bottom quintiles of different biological pathways to AF calculated in UK Biobank 19
Supplemental Table S10. Associations between genetically predicted risk of AF via differing biological pathways on left ventricular functional parameters 20
Supplemental Table S11. Sensitivity analyses for estimate of AF pathway score effects on cardioembolic stroke in GIGASTROKE 21
Supplemental Table S12. Estimates of pathways on cardioembolic stroke risk in GIGASTROKE after accounting for effects of genetic variants in pathways on ECG parameters 22
Supplemental Table S13. Estimates of pathways on cardioembolic stroke risk in GIGASTROKE after accounting for effects of genetic variants in pathways on left atrial parameters 23
Supplemental Table S14. Estimates of pathways on cardioembolic stroke risk in GIGASTROKE after accounting for effects of genetic variants in pathways on markers of LV dysfunction 24
Supplemental Figure S1. Associations between genetically predicted AF biological pathways and risk of AF in UK Biobank 25

Supplemental Figure S2. Associations between genetically predicted AF biological pathways and surface ECG PR interval adjusted for P-wave duration in UK Biobank 26
Supplemental Figure S3. Associations between comprehensive group of variants for AF risk and surface ECG parameters in UK Biobank 27
Supplemental Figure S4. Associations between comprehensive group of variants for AF risk and LA volumes and function in UK Biobank 28
Supplemental Figure S5. Associations between genetically predicted risk of AF via differing biological pathways and NT-proBNP levels in INTERVAL and IMPROVE cohorts 29
Supplemental Figure S6. Associations between comprehensive group of variants for AF risk and NT-proBNP levels 30
Supplemental Figure S7. Effects of genetically predicted AF biological pathways on high-sensitivity troponin I and T levels in different cohorts 31
Supplemental Figure S8. Associations between comprehensive group of variants for AF risk and high-sensitivity troponin I and T levels in different cohorts 32
Supplemental Figure S9. Associations between genetically predicted AF biological pathways and small-vessel ischaemic stroke 33
Supplemental Figure S10. Associations between comprehensive group of variants for AF risk and different types of ischaemic stroke in GIGASTROKE cohort 34
Supplemental Figure S11. Funnel plot for SNPs in muscle pathway for AF 35
Supplemental Figure S12. Funnel plot for SNPs in developmental pathway for AF 36
Supplemental Figure S13. Funnel plot for SNPs in ion channel pathway for AF 37
Supplemental Figure S14. Leave one out analyses for SNPs in muscle pathway for AF and cardioembolic stroke 38
Supplemental Figure S15. Leave one out analyses for SNPs in developmental pathway for AF and cardioembolic stroke 39
Supplemental Figure S16. Leave one out analyses for SNPs in ion channel pathway for AF and cardioembolic stroke 40

Supplemental Methods

Genetically-predicted mechanistic pathways associated with risk of AF

A total of 142 independent genome-wide significant variants ($P < 5 \times 10^{-8}$) were previously identified in a large AF GWAS meta-analysis, referred to as the comprehensive 142-variant set hereafter, which captures broader genetic predisposition to AF (Nielsen *et al.*, Nat Genet 2018[12]).

The authors of the same AF GWAS found 3,048 genes or transcripts for which the transcribed region overlapped at least 1 variant in the 111 loci. They prioritized biological candidate genes that:

(1) harbored a protein-altering index variant itself or were in high LD (r2> 0.80);
(2) had expression levels that were associated and colocalized with atrial fibrillation-associated variants ($P < 1.14 \times 10{-}9$ in Genotype-Tissue Expression (GTEx) consortium data);
(3) were highlighted by DEPICT (FDR < 0.05); or
(4) were nearest to the index variant in a locus.

Using these criteria, they prioritized 151 candidate genes.

The authors of the same AF GWAS[12] subsequently applied DEPICT to detect gene sets that were enriched for genes at atrial fibrillation-associated loci. Of the 14,461 gene sets tested, 889 were enriched (false discovery rate (FDR) < 0.05). The highlighted gene sets pointed to biological processes related to cardiac development and morphology along with structural remodeling of the myocardium.

They identified as functional candidates at least 18 genes likely to be involved in cardiac and skeletal muscle function and integrity (*AKAP6, CFL2, MYH6, MYH7, MYO18B, MYO1C, MYOCD, MYOT, MYOZ1, MYPN, PKP2, RBM20, SGCA, SSPN, SYNPO2L, TTN, TTN-AS, WIPF1)*;at least 13 genes likely to be involved in mediation of developmental events (*ARNT2, EPHA3, FGF5, GATA4, GTF2I, HAND2, LRRC10, NAV2, NKX2-5, PITX2, SLIT3,SOX15, TBX5*) along with genes likely to be involved in intracellular calcium handling in the heart (*CALU, CAMK2D, CASQ2, PLN*), angiogenesis (*TNFSF12, TNFSF12-TNFSF13*), hormone signaling (*CGA, ESR2, IGF1R, NR3C1, THRB*), and function of cardiac ion channels (*HCN4, KCND3, KCNH2, KCNJ5, KCNN2, KCNN3, SCN10A, SCN5A, SLC9B1*).

Further details are available within the Supplemental Materials of the original paper (Nielsen *et al.*, Nat Genet 2018[12]).

Genetic Instruments used in study

In the present study, a total of 52 independent variants from the comprehensive 142-variant set that were objectively annotated to genes within the ‘muscle function and integrity’, ‘mediation of developmental events’, and ‘function of cardiac ion channels’ gene-sets/pathways were selected in their entirety as the three pathways for subsequent testing within our study (see **Supplemental Tables S1-4**). These were non-overlapping and all were sub-sets of the comprehensive 142-variant score.

Specifically, the ‘muscle pathway’ comprised variants annotated to genes involved in cardiac and skeletal muscle function and integrity (15 genetic variants, annotated to 14 distinct genes e.g. *MYH6*,

PKP2, *TTN*). The 'developmental pathway' comprised variants annotated to genes involved in cardiac developmental events (25 genetic variants, annotated to 12 distinct genes e.g. *GATA4*, *NKX2-5*, *PITX2*, *TBX5*). The 'ion channel pathway' comprised variants annotated to genes involved in cardiac ion channels (12 genetic variants, annotated to 8 distinct genes e.g. *HCN4*, *KCNH2*, *KCNJ5*, *SCN5A*).

The AF GWAS[12] meta-analysis also identified variants annotated to genes likely to be involved in calcium handling (4 variants), angiogenesis (1 variant) and hormone signalling (4 variants). These potential pathways were not included in the present study as they were relatively non-specific and comprised multiple heterogeneous biological pathways. For example, the hormone signalling pathway identified genes associated with oestrogen receptors, thyroid receptors and insulin-like growth factor receptors. Further details are provided in **Supplemental Tables S1-4**.

Study populations and outcome definitions

Details of the datasets used in this study are listed in **Supplemental Table S5**.

Atrial fibrillation and demographics

Genetic associations with AF were generated directly in UK Biobank[66,67] in 339,189 unrelated white British participants whose genetic samples passed bioinformatic quality control using logistic regression with adjustments for sex, genotype array, and 7 principal components of population structure. We identified 30,631 cases of AF or flutter, defined using electronic healthcare records with details of definitions in **Supplemental Table S6**. Further details of phenotype codes utilised to define co-morbidities are listed in **Supplemental Table S7** with definitions of self-reported medication codes from the baseline verbal interview in UK Biobank in **Supplemental Table S8**.

ECG parameters

As with AF, genetic associations with ECG parameters were generated directly in UK Biobank using linear regression with adjustments for sex, genotype array, and 7 principal components of population structure. Resting 12-lead ECGs and automated measurements (using Cardiosoft v6 program, GE) were available in 16,508 participants, and analyses of these data were conducted after excluding individuals with AF on ECG, or AF identified as described above.

Cardiac MRI parameters

Genetic associations with cardiac MRI imaging data including LA structure and function[28] (35,658 individuals of European ancestry) and LV structure and function[29] (36,041 individuals of European ancestry), both regardless of history of AF, were based on existing summary data derived from UK Biobank. The UK Biobank cardiac MRI protocol has been reported elsewhere[68]. In brief, LA parameters were derived from automated processing algorithms; volumes were indexed to Du Bois formula calculated body surface area and cardiac volume-time curves created to derive active and passive LA emptying fractions[28]. LV parameters were provided by UK Biobank, corrected for known bias from the automated algorithm and indexed to Mosteller formula calculated body surface area[29].

NT-proBNP

Genetic associations with NT-proBNP were based on summary data including 33,043 individuals from the UK Biobank Pharma Proteomics Project (UKB-PPP)[30] and 35,559 Icelandic individuals from the deCODE + Icelandic cancer project[31]. These associations were further validated in summary data including 3,301 healthy blood donors from the INTERVAL study[32,69] and a GWAS of 3,394 individuals with cardiovascular risk factors from the IMPROVE study[33,70]. Assays for NT-proBNP included Olink (UKB-PPP, IMPROVE) and SomaScan (deCODE + ICP, INTERVAL).

High-sensitivity troponin I and T (hsTnI, hsTnT)

Genetic associations with hsTnI were based on summary data including 48,115 individuals from the Trøndelag Health Study (HUNT) and Generation Scotland Family Health Study (GS:FHS)[34] and in 14,336 individuals of diverse ancestry (89% European, 11% African) across a number of cohorts (including ARIC) but excluding individuals with baseline coronary heart disease and heart failure as described by Yang *et al*[35]. Genetic associations with hsTnT were based on summary data including 24,617 individuals (76% European, 15% African, 7% Hispanic, 3% Asian) across a number of cohorts (including ARIC, CHS, MESA) but excluding individuals with baseline coronary disease and heart failure[35].

Ischaemic stroke

Genetic associations with stroke outcomes were based on summary data from the GIGASTROKE GWAS meta-analysis[36]. Contributing studies defined the aetiology of stroke as per the TOAST (Trial of Org in 10172 Acute Stroke Treatment) classification system[37]. In the present study, we examined associations with cardioembolic stroke (10,804 European ancestry; 12,790 multi-ancestry) as a downstream consequence of AF, and small-vessel stroke as a negative control due to its different risk factor profile (6,811 European; 13,620 multi-ancestry).

Statistical analysis

Primary analyses

Ratio estimates for each individual variant were determined by dividing the gene-outcome association (for each outcome of interest as described above) by the association with AF (taken from the published GWAS for AF[12] after exclusion of UK Biobank participants to reduce the potential for bias). For each specific pathway, the individual variant ratio estimates were then combined using fixed-effects (assuming a single common effect) inverse variance weighted methods, with resulting estimates corresponding to a unit increase in log-odds risk of AF (approximately a 2.7-fold higher risk).

Heterogeneity between estimates from different pathways was assessed using Cochran’s Q statistic and Wald tests to examine pair-wise differences between individual pathways. Allowance was made for testing multiple hypotheses, namely the three pathways and 142-variant score within each of the various independent datasets considered, by defining robust statistical significance as $P<0.01$ and nominal statistical significance as $P<0.05$, as well as in the interpretation of the analyses.

Sensitivity analyses

We conducted sensitivity analyses to assess the robustness of the results to underlying methodological assumptions (based on a Mendelian randomisation [MR] instrumental variable framework) including: inverse variance weighted random effects (allowing for heterogeneity between instrumental variables); weighted median (in which up to 50% of the information is permitted to be from

invalid instrumental variables); weighted mode (which allows the majority of information to be invalid provided the most common MR effect estimate is a consistent estimate of the true MR effect); and MR-Egger (in which all genetic variants are permitted to be invalid instrumental variables, provided that the pleiotropic and risk factor effects of the variants are independently distributed)[71,72]. MR estimates from each pathway were also estimated after exclusion of outlier variants identified using the MR-PRESSO outlier detection method[73]. 'Leave-one-out' analyses were performed to test whether the effects of the pathways on cardioembolic stroke were sensitive to individual variants. Additional post-hoc analyses were also undertaken to estimate the direct effects of the pathways on outcomes showing heterogeneity between pathways (i.e. cardioembolic stroke), independent of their associations with ECG parameters, LA parameters, and markers of LV dysfunction. To further confirm that the scores were unrelated to conventional risk factors, within UK Biobank, participant-level weighted AF risk scores were calculated for each of the pathways and the characteristics of the top and bottom quintiles of score compared.

All analyses were performed using SAS (v 9.4, SAS Institute) and R (v 4.3.1, The R Foundation for Statistical Computing).

Supplemental Results

Sensitivity Analyses - Investigations for pleiotropic effects within the pathways

Where heterogeneity was observed between pathways for the outcome of cardioembolic stroke, other statistical sensitivity analyses were carried out to further investigate potential pleiotropic effects of the genetic variants constituting the AF pathways. There was no evidence of heterogeneity between the individual variant effects in any of the individual pathways (all $P > 0.05$).

However, for completeness, multiplicative random effects inverse variance weighted methods were used to account for potential heterogeneity (**Supplemental Table S11**). These had no material impact on the statistical significance of the estimated effect of any individual pathway, albeit with weaker evidence of heterogeneity between pathways for cardioembolic stroke (*P* for heterogeneity European ancestry = 0.13; *P* for heterogeneity multi-ancestry = 0.04). Multiplicative random-effects analyses across all intermediate phenotypes suggested that the most robust differences between the pathways were for PR interval, LA passive emptying fraction, and NT-proBNP across all cohorts.

The weighted median and weighted mode MR methods similarly gave comparable results to the primary analyses for cardioembolic stroke (**Supplemental Table S11**). Egger bias estimates of the effects of variants within the different AF pathways on stroke risks showed no evidence of significant directional pleiotropy, and no genetic variants within the different scores were identified as outlying genetic variants using MR-PRESSO.

Overall, there was no evidence of pleiotropy and effect estimates appeared robust to instrumental variable assumptions.

Supplemental Table S1. Genetic variants included in 142-variant set for comprehensive AF risk

rsID	Gene	Risk Allele	Ref Allele	Effect Size	SE
rs10006327	*SLC9B1*	C	T	0.0357	0.0079
rs10213171	*ARHGAP10*	G	C	0.0822	0.0155
rs10458660	*C10orf11*	G	A	0.0551	0.0102
rs10465885	*GJA5*	C	T	0.0262	0.0079
rs10520260	*HAND2,HAND2-AS1*	A	G	0.0425	0.0086
rs10741807	*NAV2*	T	C	0.0679	0.0093
rs10749053	*RBM20*	T	C	0.0533	0.0116
rs10753933	*PPFIA4*	T	G	0.0529	0.0080
rs10773657	*HIP1R*	C	A	0.0649	0.0120
rs10804493	*PHLDB2,PLCXD2*	A	G	0.0507	0.0083
rs10821415	*C9orf3*	A	C	0.0768	0.0080
rs10873298	*IRF2BPL*	C	T	0.0295	0.0082
rs11125871	*USP34*	C	T	0.0322	0.0081
rs11156751	*AKAP6*	C	T	0.0677	0.0092
rs11264280	*KCNN3*	T	C	0.1258	0.0085
rs114904067	*PITX2*	G	A	0.0526	0.0268
rs11590635	*AGBL4*	A	G	0.1731	0.0306
rs11598047	*NEURL1*	G	A	0.1498	0.0104
rs11614818	*NACA*	C	T	0.0327	0.0082
rs11658278	*ZPBP2,GSDMB,ORMD*	T	C	0.0410	0.0079
rs11773845	*CAV1,CAV2*	A	C	0.0924	0.0080
rs117984853	*UST*	T	G	0.1064	0.0144
rs118159104	*RPL3L*	G	T	0.1784	0.0412
rs12188351	*SLIT3*	A	G	0.0784	0.0169
rs12245149	*REEP3,NRBF2*	C	A	0.0376	0.0079
rs12426679	*PHLDA1*	C	T	0.0309	0.0079
rs12604076	*CYTH1,USP36*	T	C	0.0369	0.0078
rs12648245	*HAND2,HAND2-AS1*	T	C	0.0995	0.0152
rs1278493	*PPP2R3A*	G	A	0.0327	0.0080
rs12809354	*PKP2*	C	T	0.0553	0.0111
rs12908004	*ARNT2*	G	A	0.0696	0.0106
rs13195459	*HSF2*	G	A	0.0637	0.0083
rs133902	*MYO18B*	T	C	0.0478	0.0082
rs138311480	*PITX2*	C	T	0.1196	0.0397
rs140185678	*RPL3L*	A	G	0.1439	0.0286
rs1458038	*FGF5*	T	C	0.0406	0.0085
rs146518726	*MIR6500*	A	G	0.1709	0.0235
rs147301839	*GCOM1/MYZAP*	C	A	0.3406	0.0617
rs1532170	*PITX2*	G	A	0.0357	0.0084
rs1545300	*KCND3*	C	T	0.0500	0.0086

rsID	Gene	Risk Allele	Ref Allele	Effect Size	SE
rs1563304	*WNT3*	T	C	0.0669	0.0112
rs17005647	*FRMD4B*	T	C	0.0342	0.0082
rs17380837	*SSPN*	C	T	0.0439	0.0086
rs1957021	*AKAP6*	C	T	0.0489	0.0094
rs2012809	*SLC27A6*	G	A	0.0500	0.0116
rs2031522	*CGA*	A	G	0.0441	0.0081
rs2040862	*WNT8A,NPY6R,MYOT*	T	C	0.1025	0.0103
rs2274115	*LHX3*	G	A	0.0526	0.0091
rs2288327	*TTN,MIR548N,FKBP*	G	A	0.0825	0.0105
rs2291437	*LINC00477*	G	T	0.0931	0.0121
rs2359171	*ZFHX3*	A	T	0.1577	0.0101
rs244017	*PITX2*	T	G	0.0189	0.0101
rs2540949	*CEP68*	A	T	0.0625	0.0080
rs2738413	*SYNE2,MIR548AZ,E*	A	G	0.0704	0.0079
rs2759301	*ARNT2*	A	G	0.0420	0.0079
rs2834618	*LINC01426*	T	G	0.1010	0.0133
rs28387148	*GYPC*	T	C	0.0872	0.0136
rs284277	*CASZ1*	C	A	0.0432	0.0082
rs28439930	*NKX2-5*	G	C	0.0409	0.0080
rs2860482	*NACA*	A	C	0.0476	0.0091
rs2885697	*SCMH1*	G	T	0.0403	0.0082
rs3176326	*CDKN1A,PANDAR,PI*	G	A	0.0680	0.0101
rs337705	*KCNN2*	G	T	0.0583	0.0081
rs34080181	*LRIG1,SLC25A26*	G	A	0.0371	0.0083
rs34969716	*KDM1B,DEK*	A	G	0.0651	0.0094
rs35005436	*GTF2I,LOC1019269*	C	T	0.0546	0.0119
rs35176054	*NEURL1*	A	T	0.1281	0.0117
rs35544454	*ERBB4*	A	T	0.0633	0.0103
rs35569628	*CUL4A*	T	C	0.0465	0.0095
rs35620480	*GATA4*	C	A	0.0510	0.0110
rs3820888	*SPATS2L*	C	T	0.0599	0.0080
rs3853445	*PITX2*	T	C	0.1531	0.0090
rs3951016	*SLC35F1,PLN*	A	T	0.0723	0.0079
rs4073778	*CASQ2*	A	C	0.0493	0.0080
rs422068	*MYH6,MYH7*	C	T	0.0246	0.0084
rs464901	*TUBA8*	T	C	0.0418	0.0086
rs4871397	*FBXO32*	G	C	0.0829	0.0164
rs4935786	*SORL1*	T	A	0.0362	0.0095
rs4951258	*NUCKS1,SLC41A1*	A	G	0.0344	0.0079
rs4963776	*LINC00477*	G	T	0.0808	0.0104
rs4965430	*IGF1R*	C	G	0.0397	0.0081
rs4999127	*KCNN3*	A	G	0.0800	0.0119
rs55693294	*NEURL1*	T	C	0.0446	0.0177

rsID	Gene	Risk Allele	Ref Allele	Effect Size	SE
rs55734480	*DGKB*	A	G	0.0419	0.0094
rs55985730	*OPN1SW,CALU*	G	T	0.0782	0.0177
rs56181519	*WIPF1*	C	T	0.0576	0.0091
rs56201652	*CDK6*	G	A	0.0563	0.0089
rs577676	*LINC01142*	C	T	0.0977	0.0079
rs60212594	*SYNPO2L,NUDT13,M*	G	C	0.1272	0.0113
rs60902112	*XXYLT1*	T	C	0.0388	0.0093
rs61501369	*PITX2*	T	C	0.0788	0.0096
rs62521286	*FBXO32*	G	A	0.1131	0.0161
rs6462079	*CREB5*	A	G	0.0519	0.0090
rs6560886	*FBRSL1*	C	T	0.0510	0.0109
rs6580277	*NR3C1*	G	A	0.0759	0.0094
rs6596717	*LOC102467213*	C	A	0.0356	0.0080
rs6689306	*KCNN3*	A	G	0.0440	0.0081
rs67249485	*PITX2*	T	A	0.3422	0.0094
rs6747542	*GMCL1,ANXA4*	T	C	0.0602	0.0079
rs6771054	*EPHA3*	T	C	0.0405	0.0081
rs6790396	*SCN10A,SCN5A*	G	C	0.0604	0.0081
rs67969609	*TEX41*	G	C	0.0764	0.0147
rs6829664	*CAMK2D*	G	A	0.0638	0.0090
rs6850025	*PITX2*	A	G	0.1536	0.0182
rs6891790	*NKX2-5*	G	T	0.0716	0.0090
rs6994744	*PTK2*	C	A	0.0330	0.0079
rs7096385	*SIRT1,MYPN*	T	C	0.0680	0.0152
rs71454237	*LRRC10*	G	A	0.0613	0.0100
rs7170477	*HERC1*	A	G	0.0389	0.0085
rs7225165	*YWHAE,CRK,MYO1C*	G	A	0.0744	0.0135
rs72700114	*LINC01142*	C	G	0.1698	0.0154
rs72700118	*LINC01142*	A	C	0.1162	0.0120
rs72811294	*MYOCD*	G	C	0.0658	0.0126
rs72926475	*REEP1*	G	A	0.0548	0.0121
rs73041705	*THRB*	T	C	0.0465	0.0088
rs73241997	*CFL2*	T	C	0.0622	0.0109
rs73366713	*ATXN1*	G	A	0.0923	0.0120
rs7373065	*SCN10A, SCN5A*	T	C	0.1813	0.0299
rs7374540	*SCN10A, SCN5A*	A	C	0.0270	0.0080
rs74022964	*HCN4*	T	C	0.1079	0.0106
rs74884082	*DPF3*	C	T	0.0461	0.0093
rs7508	*ASAH1*	A	G	0.0711	0.0088
rs7529220	*HSPG2*	C	T	0.0686	0.0116
rs7578393	*KIF3C*	T	C	0.0615	0.0103
rs76097649	*KCNJ5*	A	G	0.0988	0.0150
rs7612445	*GNB4*	T	G	0.0428	0.0099

rsID	Gene	Risk Allele	Ref Allele	Effect Size	SE
rs7650482	*CAND2*	G	A	0.0668	0.0082
rs7687819	*PITX2*	A	G	0.0200	0.0094
rs77316573	*RPL3L*	T	C	0.0383	0.0107
rs775498	*LRRC10*	G	A	0.0362	0.0088
rs7789146	*KCNH2*	G	A	0.0568	0.0103
rs7834729	*XPO7*	G	T	0.0587	0.0123
rs79187193	*GJA5*	G	A	0.1136	0.0179
rs79399769	*PITX2*	C	T	0.1177	0.0296
rs8088085	*MEX3C*	A	C	0.0388	0.0079
rs876727	*ZFHX3*	T	G	0.0745	0.0101
rs883079	*TBX5*	T	C	0.0872	0.0087
rs9401451	HSF2	G	A	0.0703	0.0129
rs9506925	LINC00540, LINC00	T	C	0.0365	0.0090
rs9899183	TNFSF12	T	C	0.0432	0.0090
rs9953366	SMAD7	C	T	0.0442	0.0087
rs9963878	MEX3C	C	T	0.0695	0.0142

Genetic variants considered significant included in the 142-variant set of genetic variants for AF risk taken from Nielsen *et al.* 2018 AF GWAS. Effect size is expressed as log odds risk of AF per copy of risk allele and excludes UK Biobank. SE: standard error.

Supplemental Table S2. Genetic variants included in muscle pathway for AF

rsID	Gene	Risk Allele	Ref Allele	Effect Size	SE
rs10749053	*RBM20*	T	C	0.0533	0.0116
rs11156751*	*AKAP6*	C	T	0.0677	0.0092
rs12809354	*PKP2*	C	T	0.0553	0.0111
rs133902	*MYO18B*	T	C	0.0478	0.0082
rs17380837	*SSPN*	C	T	0.0439	0.0086
rs1957021	*AKAP6*	C	T	0.0489	0.0094
rs2040862	*MYOT*	T	C	0.1025	0.0103
rs2288327	*TTN*	G	A	0.0825	0.0105
rs422068	*MYH6, MYH7*	C	T	0.0246	0.0084
rs56181519	*WIPF1*	C	T	0.0576	0.0091
rs60212594	*SYNPO2L*	G	C	0.1272	0.0113
rs7096385	*SIRT1, MYPN*	T	C	0.0680	0.0152
rs7225165	*YWHAE, CRK, MYO1C*	G	A	0.0744	0.0135
rs72811294	*MYOCD*	G	C	0.0658	0.0126
rs73241997	*CFL2*	T	C	0.0622	0.0109

Genetic variants annotated to the ‘cardiac muscle’ pathway in AF GWAS based on functional pathway analyses. Effect size is expressed as log odds risk of AF per copy of risk allele. SE: standard error. All genetic variants were available in UKB analyses (AF, ECG, cardiac MRI, NT-proBNP), deCODE+ICP NT-proBNP analyses, HUNT + GS:FHS troponin, and HERMES heart failure analyses. Variants denoted by * were not available in primary GIGASTROKE analysis for cardioembolic stroke in those of European ancestry.

Supplemental Table S3. Genetic variants included in developmental pathway for AF

rsID	Gene	Risk Allele	Ref Allele	Effect Size	SE
rs10520260	*HAND2, HAND2-AS1*	A	G	0.0425	0.0086
rs10741807	*NAV2*	T	C	0.0679	0.0093
rs114904067	*PITX2*	G	A	0.0526	0.0268
rs12188351	*SLIT3*	A	G	0.0784	0.0169
rs12648245*	*HAND2, HAND2-AS1*	T	C	0.0995	0.0152
rs12908004	*ARNT2*	G	A	0.0696	0.0106
rs138311480*	*PITX2*	C	T	0.1196	0.0397
rs1458038	*FGF5*	T	C	0.0406	0.0085
rs1532170	*PITX2*	G	A	0.0357	0.0084
rs244017	*PITX2*	T	G	0.0189	0.0101
rs2759301	*ARNT2*	A	G	0.0420	0.0079
rs28439930	*NKX2-5*	G	C	0.0409	0.0080
rs35005436	*GTF2I, LOC1019269*	C	T	0.0546	0.0119
rs35620480	*GATA4*	C	A	0.0510	0.0110
rs3853445	*PITX2*	T	C	0.1531	0.0090
rs61501369	*PITX2*	T	C	0.0788	0.0096
rs67249485	*PITX2*	T	A	0.3422	0.0094
rs6771054	*EPHA3*	T	C	0.0405	0.0081
rs6850025	*PITX2*	A	G	0.1536	0.0182
rs6891790	*NKX2-5*	G	T	0.0716	0.009
rs71454237	*LRRC10*	G	A	0.0613	0.0100
rs7687819	*PITX2*	A	G	0.0200	0.0094
rs775498	*LRRC10*	G	A	0.0362	0.0088
rs79399769	*PITX2*	C	T	0.1177	0.0296
rs883079	*TBX5*	T	C	0.0872	0.0087

Genetic variants annotated to the ‘developmental’ pathway in AF GWAS based on functional pathway analyses. Effect size is expressed as log odds risk of AF per copy of risk allele. SE: standard error. All genetic variants available in UKB analyses (AF, ECG, cardiac MRI, NT-proBNP), deCODE+ICP NT-proBNP analyses, and HUNT + GS:FHS troponin analyses. Variants denoted by * were not available in primary GIGASTROKE analysis for cardioembolic stroke in those of European ancestry.

Supplemental Table S4. Genetic variants included in ion channel pathway for AF

rsID	Gene	Risk Allele	Ref Allele	Effect Size	SE
rs10006327	*SLC9B1*	C	T	0.0357	0.0079
rs11264280	*KCNN3*	T	C	0.1258	0.0085
rs1545300	*KCND3*	C	T	0.0500	0.0086
rs337705	*KCNN2*	G	T	0.0583	0.0081
rs4999127	*KCNN3*	A	G	0.0800	0.0119
rs6689306	*KCNN3*	A	G	0.0440	0.0081
rs6790396	*SCN10A, SCN5A*	G	C	0.0604	0.0081
rs7373065	*SCN10A, SCN5A*	T	C	0.1813	0.0299
rs7374540	*SCN10A, SCN5A*	A	C	0.0270	0.0080
rs74022964	*HCN4*	T	C	0.1079	0.0106
rs76097649	*KCNJ5*	A	G	0.0988	0.0150
rs7789146	*KCNH2*	G	A	0.0568	0.0103

Genetic variants annotated to the 'ion channel' pathway in AF GWAS based on functional pathway analyses. Effect size is expressed as log odds risk of AF per copy of risk allele. SE: standard error. All genetic variants available in UKB analyses (AF, ECG, cardiac MRI, NT-proBNP), deCODE+ICP NT-proBNP, HUNT + GS:FHS troponin, and primary GIGASTROKE analysis for cardioembolic stroke in those of European ancestry.

Supplemental Table S5. Details of datasets used in study

Cohort	Demographic Details	Phenotype	Numbers	Comments
**UK Biobank**	White British; mean age 57-59 based on subsets, 54% female	Atrial fibrillation	30,631 cases; 308,558 controls	Individual level data, case max AF dataset
		ECG parameters	16,508 individuals	Individual level data, AF excluded
		Left atrial volumes and function	35,658 individuals	Summary data (Cardiac MRI)
		Left ventricular function	36,041 individuals	Summary data (Cardiac MRI)
		NT-proBNP (Olink assay)	33,043 individuals	Summary data from UKB-PPP
**deCODE + ICP**	European (Icelandic); mean age 55, 57% female	NT-proBNP (SOMA assay)	35,559 individuals	Summary data
**INTERVAL study**	European; healthy blood donors with mean age 43, 48% female	NT-proBNP (SOMA assay)	3,301 individuals	Summary data
**IMPROVE study**	European; with >3 risk factors for CV disease and mean age 65, 52% female	NT-proBNP (Olink assay)	3,394 individuals	Summary data
**HUNT + GS:FHS**	European; median ages 59 for HUNT and 49 for GS:FHS, 55% female for HUNT and 58% female for GS:FHS	hs-Troponin I	48,115 individuals	Summary data; median hs-TnI level ~2 ng/L
**Multi-ethnic (Yang *et. al.*, 2021)**	89% European, 11% African; free from prevalent coronary heart disease or heart failure with mean age 61, ~55% female	hs-Troponin I	14,336 individuals	Summary data; mean hs-TnI level ~8 ng/L
	76% European, 15% African, 10% other; free from prevalent coronary heart disease or heart failure with mean age 67, females ~55% female	hs-Troponin T	24,617 individuals	Summary data; mean hs-TnT level ~7ng/L

Cohort	Demographic Details	Phenotype	Numbers	Comments
**GIGASTROKE**	Separate datasets for European ancestry only and multi-ethnic ancestry (84.5% European) with mean age ~69 years at time of stroke, ~54% female	Cardioembolic stroke	European ancestry: 10,804 cases; 1,234,808 controls<br>Multi-ethnic ancestry: 12,790 cases; 1,503,898 controls	Summary data; TOAST classified stroke
		Small vessel stroke	European ancestry: 6,811 cases; 1,234,808 controls<br>Multi-ethnic ancestry: 13,620 cases; 1,503,898 controls	Summary data; TOAST classified stroke

UK Biobank: AF and ECG data via UKB Application 14568; cardiac MRI data accessed via https://zenodo.org/record/5074929#.ZC2YfHbMKUk and www.broadcvdi.org from Ahlberg *et al*., *Eur Heart J* 2021;**42**:4523-4534 and Pirruccello *et al*., *Nat Commun* 2020;**11**:2254. NT-proBNP data accessed via https://www.synapse.org/#!Synapse:syn51364943/wiki/622119 from Sun *et al.*, *Nature* 2023;**622**:329-338.

DeCode+ICP: Data on NT-proBNP accessed via https://www.decode.com/summarydata/ from Ferkingstad *et al*., *Nat Genet* 2021;**53**:1712-1721.

INTERVAL: Data on NT-proBNP accessed via https://gwas.mrcieu.ac.uk/ from Sun *et al*., *Nature* 2018;**558**:73-79.

IMPROVE: Data on NT-proBNP accessed via via https://zenodo.org/record/264128#.XqwKuKhKhPY from Folkersen *et al*., *PLoS Genet* 2017;**13**:e1006706.

HUNT + GS:FHS: Data on high-sensitivity Troponin I provided to us on request by authors from Moksnes *et al*., *Hum Mol Genet* 2021;**30**:2027-2039.

Multi-ethnic high-sensitivity Troponin I/T data: Provided to us on request by authors from Yang *et al*., *Circ Genom Precis Med* 2021;**14**:e003460.

GIGASTROKE: Data on stroke subtypes accessed via https://www.ebi.ac.uk/gwas/home from Mishra *et al*., *Nature* 2022;**611**:115-123.

Supplemental Table S6. Phenotype definition of Atrial Fibrillation in UK Biobank

Field	Description	Algorithm Elements
20002	Non-cancer illness code, self-reported	Atrial Fibrillation – 1471, Atrial Flutter – 1483
20004	Operation code	Cardiac Ablation – 1553, Cardioversion – 1524
41202	Diagnoses – main ICD10	Atrial Fibrillation or Flutter – I48*
41204	Diagnoses – secondary ICD10	Atrial Fibrillation or Flutter – I48*
41200	Operative procedures – main OPCS	K22.3 Exclusion of left atrial appendage,<br>K57.1 Percutaneous transluminal ablation of atrioventricular node,<br>K57.5 Percutaneous transluminal ablation of atrial wall,<br>K62.1 Percutaneous transluminal ablation of pulmonary vein to left atrium conducting system,<br>K62.2 Percutaneous transluminal ablation of atrial wall for atrial flutter,<br>K62.3 Percutaneous transluminal ablation of conducting system of heart for atrial flutter,<br>K62.4 Percutaneous transluminal internal cardioversion,<br>K62.5 Percutaneous transluminal occlusion of left atrial appendage,<br>X50.1 Direct current cardioversion,<br>X50.2 External cardioversion
41210	Operative procedures – secondary OPCS	K22.3 Exclusion of left atrial appendage,<br>K57.1 Percutaneous transluminal ablation of atrioventricular node,<br>K57.5 Percutaneous transluminal ablation of atrial wall,<br>K62.1 Percutaneous transluminal ablation of pulmonary vein to left atrium conducting system,<br>K62.2 Percutaneous transluminal ablation of atrial wall for atrial flutter,<br>K62.3 Percutaneous transluminal ablation of conducting system of heart for atrial flutter,<br>K62.4 Percutaneous transluminal internal cardioversion,<br>K62.5 Percutaneous transluminal occlusion of left atrial appendage,<br>X50.1 Direct current cardioversion,<br>X50.2 External cardioversion
40001	Underlying (primary) cause of death: ICD10	Atrial Fibrillation or Flutter – I48*
40002	Contributory (secondary) causes of death: ICD10	Atrial Fibrillation or Flutter – I48*

*****Indicates all ICD10 sub-codes under main heading included. AF cases were defined using electronic healthcare records and had at least one diagnostic code from: national in-patient hospital episode statistics (International Statistical Classification of Diseases 10th revision [ICD-10] codes), procedure codes (national classification of interventions and procedure [OPCS-4]), national death records, or from self-reports (of ‘illness’ or ‘operation’) at baseline verbal interview.

Supplemental Table S7. Phenotype definitions of other diseases in UK Biobank

Disease	Code Type	Codes and Description
Coronary Heart Disease	ICD10	Angina – I20*
		Acute Myocardial Infarction – I21*
		Subsequent Myocardial Infarction – I22*
		Certain current complications following acute myocardial infarction – I23*
		Other acute ischaemic heart diseases – I24*
		Chronic ischaemic heart disease – I25*
	OPCS	Transluminal balloon angioplasty of coronary artery – K49*
		Other therapeutic transluminal operations on coronary artery – K50*
		Percutaneous transluminal balloon angioplasty and insertion of stent into coronary artery – K75*
		Saphenous vein graft replacement of coronary artery – K40*
		Other autograft replacement of coronary artery – K41*
		Allograft replacement of coronary artery – K42*
		Prosthetic replacement of coronary artery – K43*
		Other replacement of coronary artery – K44*
		Connection of thoracic artery to coronary artery – K45*
		Other bypass of coronary artery – K46*
	Self-reported	Illness codes - 1074, 1075
		Operation codes – 1070, 1071, 1095
Heart failure	ICD10	Heart Failure – I50*
		Hypertensive heart disease – I11.0
		Hypertensive heart and renal disease – I13.0, I13.2
	Self-reported	Illness code – 1076
Hypertension	ICD10	Essential (primary) hypertension – I10*
		Hypertensive heart disease – I11*
		Hypertensive heart and renal disease – I13*
		Secondary hypertension – I15*
	Self-reported	Illness codes – 1065, 1072
Any diabetes	ICD10	Insulin-dependent diabetes mellitus – E10*
		Non-insulin-dependent diabetes mellitus – E11*
		Other specified diabetes mellitus – E13*
		Unspecified diabetes mellitus – E14*
	Self-reported	Probable Type 1 and Type 2 Diabetes used from algorithm validated in UK Biobank data from Eastwood *et. al*, *PLOS One,* 2016.

*****Indicates all ICD10 sub-codes under main heading included.

Supplemental Table S8. Code definitions for medications

Disease	Self-reported Medication Codes from Verbal Interview (Field ID 20003)
ACE-I or ARB	1140860696, 1140860714, 1140860728, 1140881706, 1140860750, 1140860758, 1140860752, 1141150328, 1141150560, 1141151382, 1141167758, 1141167822, 1140888552, 1140860776, 1140881712, 1141170870, 1140888560, 1140860802, 1140860806, 1141188408, 1141199940, 1141200698, 1140888556, 1140860878, 1140860882, 1140860892, 1140860904, 1140860912, 1140860918, 1140923712, 1140923718, 1141164148, 1141164154, 1140916356, 1140916362, 1141179974, 1141145660, 1141145668, 1141152998, 1141153006, 1141156836, 1141156846, 1141166006, 1141172492, 1141193282, 1141193346, 1141171336, 1141171344, 1141145658
Beta-blockers	1140916342, 1140866692, 1140879822, 1140863724, 1140909368, 1141168498, 1141187780, 1140879824, 1140860250, 1140851492, 1140860244, 1140879826, 1140878098, 1140875808, 1140860192, 1140860194, 1140879834, 1140860292, 1140860294, 1140910614, 1140879842, 1140866800, 1140866804, 1141156754, 1141156808, 1141152076, 1140866766, 1140866802, 1140866782, 1140866778, 1140866798, 1140866784, 1140881722, 1140866704, 1140866712, 1140866764, 1140851556, 1141172742, 1141187048, 1140917076, 1140916868, 1140916730, 1140879830, 1140851480, 1140851576, 1140860212, 1140860220, 1140860222, 1140860230, 1140879854, 1140860304, 1140860362, 1140879866, 1140875840, 1140860382, 1140881882, 1140860380, 1140866724, 1140866726, 1140866738, 1140866756, 1140866758, 1140922930, 1140860172, 1140864410, 1140879758, 1140860232, 1140881890, 1140879760, 1141171152, 1140860434, 1140860492, 1141182904, 1141184324, 1140879762, 1140860498, 1140879818, 1140860274, 1140860402, 1140851522, 1141182968, 1140860180, 1140860266, 1140860278, 1141164276, 1141164280
Anti-arrhythmic medications (defined as any Class I or III anti-arrhythmic or non-dihydropyridine calcium-channel blocker)	1140851530, 1140888478, 1140866584, 1140866568, 1141157186, 1140866574, 1140866576, 1140888570, 1140866654, 1140888482, 1140866612, 1140888502, 1140866516, 1140881726, 1141166600, 1141187440, 1140888510, 1140866460, 1140866466, 1140866484, 1140866546, 1140866554, 1140881692, 1140926954, 1141150926, 1141169096, 1141169710, 1141184390, 1141187056, 1141187774, 1140879806, 1140851730, 1140861128, 1140861130, 1140861136, 1140861138, 1140926780, 1140861166, 1140911698, 1140917428, 1140917452, 1140923618, 1141151474, 1141153454, 1141156656, 1141157136, 1141167832, 1141171804, 1141174684, 1141175224, 1141180238, 1141185444
Dihydropyridine calcium-channel blockers	1140861088, 1140851790, 1141150538, 1141157140, 1140861090, 1140881702, 1140861106, 1140861110, 1140861114, 1140861120, 1140911088, 1140916930, 1140923572, 1141187962, 1140926188, 1140927934, 1140927940, 1141145870, 1141150500, 1141152600, 1141162546, 1141166752, 1141169730, 1141173766, 1141188936, 1141190548, 1140879810, 1140861176, 1140861190, 1140861194, 1140879802, 1140861202, 1141200400, 1140872472, 1140872568, 1140926966, 1140888646, 1140928212, 1141199858, 1141188152, 1141187094, 1141188576, 1141188836, 1141188920, 1141190160, 1141200782, 1141201814, 1140928226, 1140928234, 1140861276, 1140851794, 1140861282
Statin	1140861958, 1140881748, 1141200040, 1141188146, 1140888594, 1140864592, 1140888648, 1140861970, 1140910632, 1140910654, 1141146234, 1141146138, 1141192410, 1141192414
Aspirin or Antiplatelet	1140868226, 1140856212, 1140856214, 1140856220, 1140856310, 1140856312, 1140856314, 1140861800, 1140861804, 1140861808, 1140863514, 1140864860, 1140882190, 1140882192, 1140909480, 1140909772, 1140917408, 1140925942, 1141151924, 1141167026, 1141177826, 1140856224 1140861778, 1140851930, 1140861780, 1140861790, 1140911710, 1141163324, 1141163328, 1141168318, 1141168322
Anticoagulant	1140881842, 1140861506, 1140861574, 1140861578, 1140888204, 1140861584, 1140861588, 1140861594, 1140888206, 1140861602, 1140861604, 1140926444, 1140926360, 1141171364, 1141171374, 1141189054, 1141189210, 1141189212, 1140864212, 1140864214, 1140888266, 1140910832, 1140861696, 1140864122, 1141164760, 1140909770, 1140861698, 1140861702, 1140861704

Supplemental Table S9. Characteristics of top and bottom quintiles of different biological pathways to AF calculated in UK Biobank

	Muscle pathway AF risk score		Developmental pathway AF risk score		Ion channel pathway AF risk score	
**UK Biobank - 339,189 unrelated White British participants**	**Bottom 20%**	**Top 20%**	**Bottom 20%**	**Top 20%**	**Bottom 20%**	**Top 20%**
Number of participants	67,832	67,830	67,829	67,828	67,834	67,827
Age at recruitment / years	57 ± 8	57 ± 8	57 ± 8	57 ± 8	57 ± 8	57 ± 8
Female sex	36,294 (53.5)	36,355 (53.6)	36,315 (53.5)	36,546 (53.8)	36,350 (53.6)	36,287 (53.5)
Body mass index / kg m^{-2}	27.4 ± 5	27.4 ± 5	27.4 ± 5	27.4 ± 5	27.4 ± 5	27.4 ± 5
Coronary heart disease	8,330 (12.3)	8,579 (12.7)	8,103 (12)	8,857 (13.1)	8,248 (12.2)	8,663 (12.8)
Heart failure	2,614 (3.9)	2,906 (4.3)	2,541 (3.8)	3,202 (4.7)	2,636 (3.9)	3,007 (4.4)
Hypertension	29,939 (44.1)	29,798 (43.9)	29,215 (43.1)	30,422 (44.9)	29,726 (43.8)	29,808 (44)
Any diabetes	5,911 (8.7)	6,055 (8.9)	5,824 (8.6)	5,970 (8.8)	5,978 (8.8)	5,793 (8.5)
Atrial fibrillation	5,057 (7.5)	7,292 (10.8)	4,597 (6.8)	8,492 (12.5)	5,165 (7.6)	7,361 (10.9)
ACE-I or ARB	9,636 (14.2)	9,468 (14.0)	9,323 (13.7)	9,855 (14.5)	9,528 (14.1)	4,493 (14.0)
Beta-blockers	4,835 (7.1)	5,073 (7.5)	4,690 (6.9)	5,252 (7.7)	4,793 (7.1)	4,928 (7.3)
Anti-arrhythmic medications	760 (1.1)	957 (1.4)	700 (1.0)	1,083 (1.6)	781 (1.2)	945 (1.4)
Dihydropyridine calcium-channel blockers	4,265 (6.3)	4,308 (6.4)	4,123 (6.1)	4,394 (6.5)	4,242 (6.3)	4,191 (6.2)
Statin	11,328 (16.7)	11,302 (16.7)	11,107 (16.4)	11,506 (16.7)	11,321 (16.7)	11,200 (16.5)
Aspirin or antiplatelet	9,537 (14.1)	9,522 (14.0)	9,248 (13.6)	9,596 (14.1)	9,268 (13.7)	9,440 (13.9)
Anticoagulant	671 (1.0)	1063 (1.6)	651 (1.0)	1,219 (1.8)	707 (1.0)	1,015 (1.5)

ACE-I: Angiotensin converting enzyme inhibitor; ARB: Angiotensin receptor blocker. Demographics of top versus bottom quintiles of individual weighted genetic risk score for atrial fibrillation (AF) for each biological pathway. Continuous variables shown as mean ± standard deviation and discrete variables as number (%). For definitions of disease phenotypes and self-reported baseline medications see **Supplemental Tables S6-8**.

Supplemental Table S10. Associations between genetically predicted risk of AF via differing biological pathways on left ventricular functional parameters

	Ejection Fraction		Indexed stroke volume	
**Pathway**	**Effect as % (95% CI)**	***P***	**Effect in ml/m^2 (95% CI)**	***P***
**UKB GWAS***				
Muscle AF	0.36 (-0.03 to 0.75)	0.07	-0.65 (-1.18 to -0.12)	0.02
Developmental AF	-0.18 (-0.39 to 0.03)	0.09	-0.23 (-0.51 to 0.05)	0.10
Ion Channel AF	-0.05 (-0.43 to 0.33)	0.80	0.09 (-0.42 to 0.60)	0.72
	P for heterogeneity	0.06	*P* for heterogeneity	0.14

AF: atrial fibrillation; CI: confidence interval; UKB GWAS: UK Biobank Genome Wide Association Study

Effect sizes expressed as per unit higher log odds genetically predicted risk of AF (via different pathway) and calculated from summary data using inverse variance weighted methods. Heterogeneity tested using Cochran's Q statistic.

*Summary GWAS data from Pirruccello *et al.* in 36,041 participants with cardiac magnetic resonance imaging data

Supplemental Table S11. Sensitivity analyses for estimate of AF pathway score effects on cardioembolic stroke in GIGASTROKE

	**Muscle pathway** *per log odds higher risk of AF*		**Developmental pathway** *per log odds higher risk of AF*		**Ion channel pathway** *per log odds higher risk of AF*	
	**OR* (95% CI)**	***P***	**OR* (95% CI)**	***P***	**OR* (95% CI)**	***P***
**GIGASTROKE – 10804 cases**						
IVW random effects	2.13 (1.78 – 2.55)	4×10^{-16}	2.13 (1.97 – 2.32)	3×10^{-73}	1.70 (1.38 – 2.09)	5×10^{-7}
Weighted Median MR estimate	2.24 (1.76 – 2.85)	6×10^{-11}	2.17 (1.94 – 2.42)	2×10^{-43}	1.71 (1.37 – 2.14)	2×10^{-6}
Weighted Mode MR estimate	2.19 (1.64 – 2.94)	1×10^{-4}	2.15 (1.94 – 2.38)	9×10^{-13}	1.72 (1.35 – 2.19)	1×10^{-3}
MR-Egger estimate	2.30 (1.40 – 3.77)	1×10^{-3}	2.22 (1.97 – 2.50)	$<3 \times 10^{-308}$	2.13 (1.33 – 3.41)	2×10^{-3}
Egger-intercept test	-	*0.74*	-	*0.36*	-	*0.30*

Sensitivity analyses for main atrial fibrillation (AF) pathway scores on risk of cardioembolic stroke in GIGASTROKE. Odds ratios (OR) and 95% Confidence Intervals (CI) presented per log odds higher risk of genetically predicted AF but (*) not applicable to Egger-intercept test.

Supplemental Table S12. Estimates of pathways on cardioembolic stroke risk in GIGASTROKE after accounting for effects of genetic variants in pathways on ECG parameters

	**Muscle pathway**		**Developmental pathway**		**Ion channel pathway**	
	**OR* (95% CI)**	***P***	**OR* (95% CI)**	***P***	**OR* (95% CI)**	***P***
**GIGASTROKE – 10,804 cases**						
Unadjusted estimate for pathway per log-odds increase in AF	2.13 (1.80 – 2.51)	8×10^{-19}	2.13 (1.97 – 2.32)	3×10^{-73}	1.70 (1.45 – 1.99)	4×10^{-11}
Estimate for pathway per log-odds increase in AF conditional on the SNP effects on:						
ECG P-wave duration	2.12 (1.75 – 2.56)	5×10^{-6}	2.09 (1.89 – 2.29)	4×10^{-12}	1.80 (1.44 – 2.25)	8×10^{-4}
ECG PR interval	1.98 (1.62 – 2.44)	3×10^{-5}	2.14 (1.98 – 2.30)	4×10^{-14}	1.80 (1.44 – 2.26)	9×10^{-4}
ECG QRS duration	2.29 (1.85 – 2.85)	7×10^{-6}	2.14 (1.99 – 2.31)	3×10^{-14}	1.92 (1.53 – 2.39)	4×10^{-4}
ECG QTc interval	2.08 (1.72 – 2.53)	8×10^{-6}	2.15 (1.98 – 2.31)	3×10^{-14}	1.64 (1.36 – 1.98)	9×10^{-4}
ECG Ventricular rate	2.11 (1.74 – 2.55)	5×10^{-6}	2.13 (1.98 – 2.30)	4×10^{-14}	1.66 (1.28 – 2.14)	5×10^{-3}
All of the above	*2.10 (1.64 – 2.70)*	*4×10^{-4}*	*2.01 (1.79 – 2.27)*	*8×10^{-9}*	*1.74 (1.23 – 2.48)*	*0.04*

Effects on cardioembolic stroke in GIGASTROKE dataset via different pathways for atrial fibrillation (AF) after accounting for their genetic associations with individual and then combined ECG parameters. Effects on cardioembolic stroke risk shown as odds ratio (OR) and 95% confidence intervals (CI) per unit increase in genetically-predicted log-odds risk of AF.

Supplemental Table S13. Estimates of pathways on cardioembolic stroke risk in GIGASTROKE after accounting for effects of genetic variants in pathways on left atrial parameters

	**Muscle pathway**		**Developmental pathway**		**Ion channel pathway**	
	**OR* (95% CI)**	***P***	**OR* (95% CI)**	***P***	**OR* (95% CI)**	***P***
**GIGASTROKE – 10,804 cases**						
Unadjusted estimate for pathway per log-odds increase in AF	2.13 (1.80 – 2.51)	8×10^{-19}	2.13 (1.97 – 2.32)	3×10^{-73}	1.70 (1.45 – 1.99)	4×10^{-11}
Estimate for pathway per log-odds increase in AF conditional on the SNP effects on:						
Maximum LA volume	2.12 (1.71 – 2.64)	2×10^{-5}	2.14 (1.97 – 2.33)	2×10^{-13}	1.73 (1.39 – 2.15)	1×10^{-3}
Minimum LA volume	2.17 (1.70 – 2.77)	5×10^{-5}	2.13 (1.96 – 2.32)	3×10^{-13}	1.68 (1.34 – 2.11)	2×10^{-3}
Passive phase LA EF	2.12 (1.71 – 2.64)	2×10^{-5}	2.14 (1.97 – 2.33)	2×10^{-13}	1.67 (1.32 – 2.10)	2×10^{-3}
Active phase LA EF	2.15 (1.69 – 2.74)	5×10^{-5}	2.14 (1.94 – 2.35)	2×10^{-12}	1.61 (1.25 – 2.09)	7×10^{-3}
All of the above	*2.10 (1.61 – 2.71)*	3×10^{-4}	*2.26 (2.01 – 2.54)*	3×10^{-9}	*1.49 (1.23 – 1.82)*	*0.01*

Effects on cardioembolic stroke in GIGASTROKE dataset via different pathways for atrial fibrillation (AF) after accounting for their genetic associations with individual and then combined parameters of left atrial (LA) size and function (ejection fraction; EF). Effects on cardioembolic stroke risk shown as odds ratio (OR) and 95% confidence intervals (CI) per unit increase in genetically-predicted log-odds risk of AF.

Supplemental Table S14. Estimates of pathways on cardioembolic stroke risk in GIGASTROKE after accounting for effects of genetic variants in pathways on markers of LV dysfunction

	Muscle pathway		Developmental pathway		Ion channel pathway	
	**OR* (95% CI)**	***P***	**OR* (95% CI)**	***P***	**OR* (95% CI)**	***P***
**GIGASTROKE – 10,804 cases**						
Unadjusted estimate for pathway per log-odds increase in AF	2.13 (1.80 – 2.51)	8×10^{-19}	2.13 (1.97 – 2.32)	3×10^{-73}	1.70 (1.45 – 1.99)	4×10^{-11}
Estimate for pathway per log-odds increase in AF conditional on the SNP effects on:						
NT-proBNP (UKB-PPP)	2.16 (1.70 – 2.76)	4×10^{-5}	2.14 (1.98 – 2.31)	4×10^{-14}	1.68 (1.36 – 2.08)	1×10^{-3}
NT-proBNP (deCODE+ICP)	2.11 (1.65 – 2.70)	7×10^{-5}	2.16 (2.01 – 2.32)	9×10^{-15}	1.61 (1.26 – 2.06)	5×10^{-3}
LVEF (UKB)	2.11 (1.74 – 2.56)	7×10^{-6}	2.15 (2.00– 2.33)	4×10^{-14}	1.76 (1.36 – 2.23)	3×10^{-3}

Effects on cardioembolic stroke in GIGASTROKE dataset via different pathways for atrial fibrillation (AF) after accounting for their genetic associations with N-terminal pro B-type natriuretic peptide (NT-proBNP) via estimates from UK Biobank Pharma Proteomics Projects (UKB-PPP) and deCODE + Icelandic Cancer Project, and left ventricular ejection fraction (LVEF) using cardiac MRI estimates from UK Biobank. Effects on cardioembolic stroke risk shown as odds ratio (OR) and 95% confidence intervals (CI) per unit increase in genetically-predicted log-odds risk of AF.

Supplemental Figure S1. Associations between genetically predicted AF biological pathways and risk of AF in UK Biobank

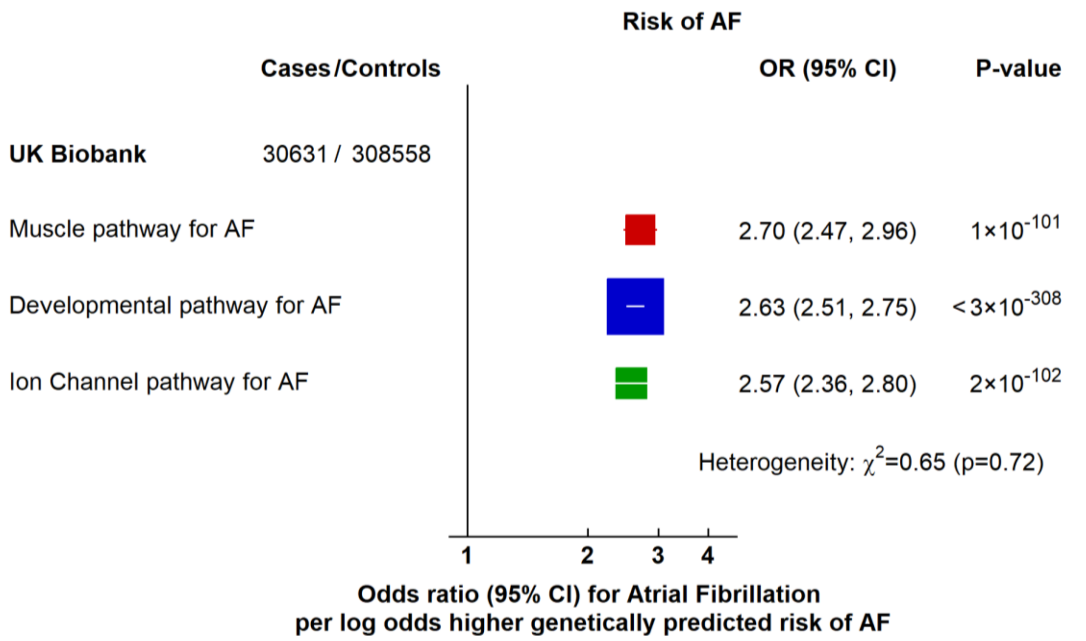

Odds ratios (OR) for atrial fibrillation (AF) in 339,189 participants in UK Biobank of White British ancestry. Boxes represent effect estimates per log odds higher genetically predicted risk of AF (defined in **Supplemental Methods** and **Supplemental Table S6**), with their size inversely proportional to variance and solid lines representing 95% confidence intervals (CI). ORs were calculated using inverse variance weighted methods, with heterogeneity tested using Cochran's Q statistic.

Supplemental Figure S2. Associations between genetically predicted AF biological pathways and surface ECG PR interval adjusted for P-wave duration in UK Biobank

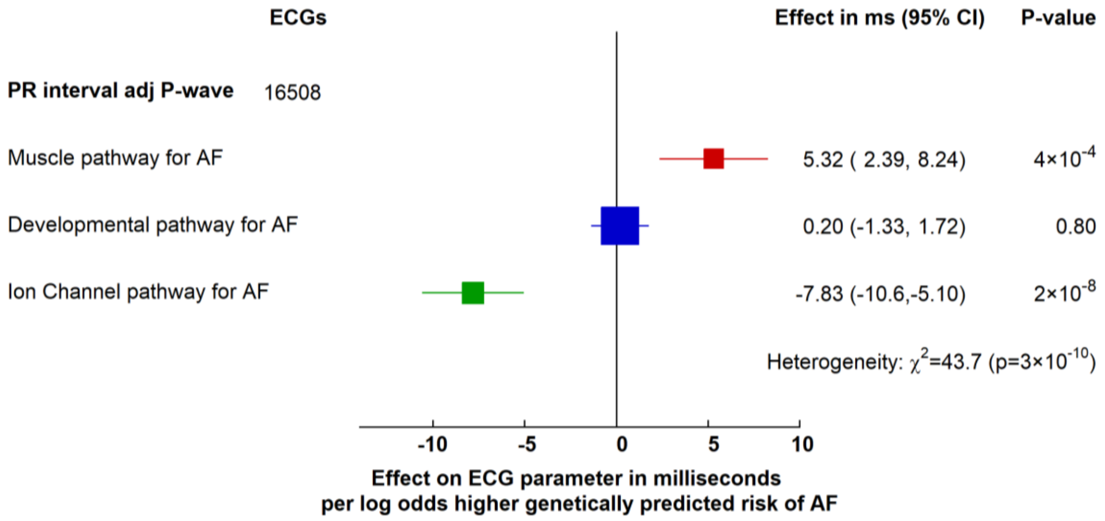

Effect of genetically predicted AF biological pathways on surface ECG PR interval adjusted for P-wave duration (in milliseconds) in a UK Biobank cohort of 16,508 participants without a history of AF. Boxes represent point estimates of effect per log-odds higher genetically predicted risk of AF, with their size inversely proportional to variance and solid lines representing 95% confidence intervals (CI). Effect sizes were calculated using inverse variance weighted methods, and heterogeneity tested using Cochran's Q statistic.

Supplemental Figure S3. Associations between comprehensive group of variants for AF risk and surface ECG parameters in UK Biobank

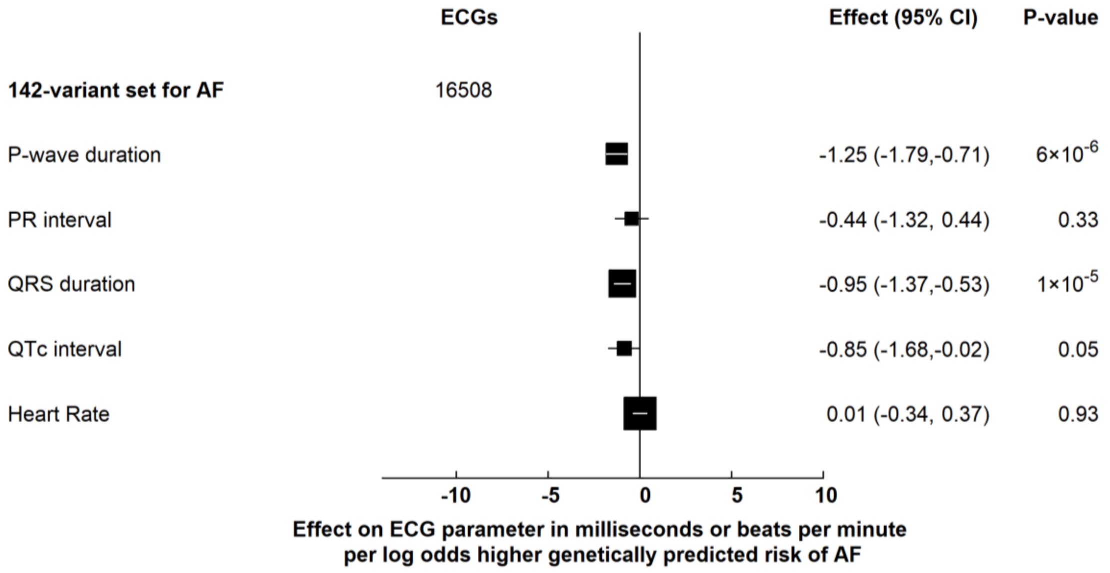

Effect of comprehensive atrial fibrillation (AF) risk predicting group of 142 independent genetic variants on ECG parameters (in milliseconds) or heart rate (in beats per minute) in UK Biobank cohort of 16,508 participant ECGs excluding any history of AF. Boxes represent point estimates of effect per log odds higher genetically predicted risk of AF, with their size inversely proportional to variance and solid lines representing 95% confidence intervals (CI). Effect sizes were calculated using inverse variance weighted methods. For comparison to pathway scores see **Figure 2** in main paper.

Supplemental Figure S4. Associations between comprehensive group of variants for AF risk and LA volumes and function in UK Biobank

A.

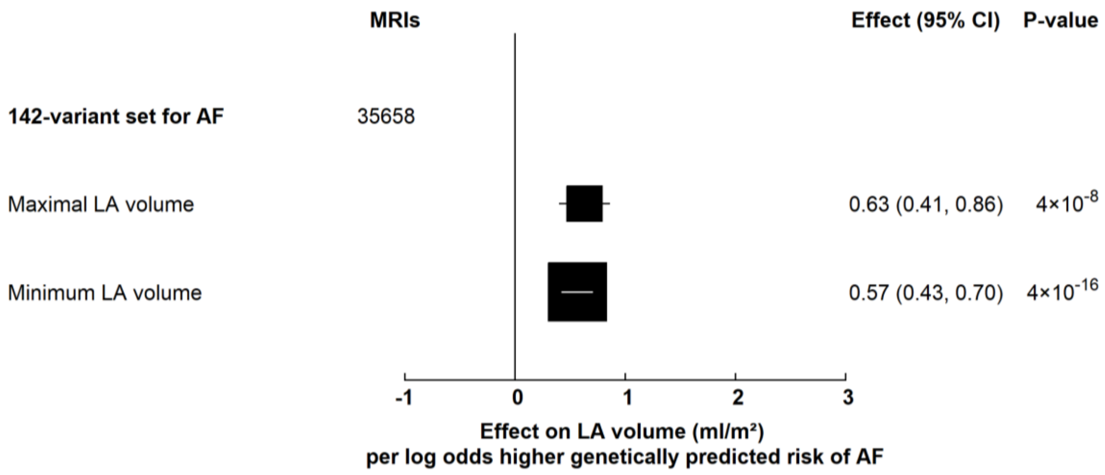

B.

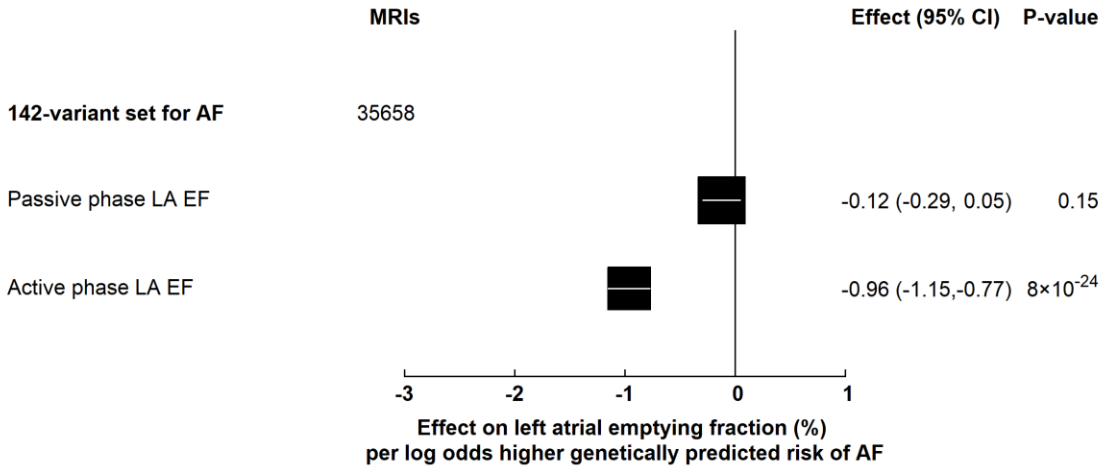

Effect of comprehensive atrial fibrillation (AF) risk predicting group of 142 independent genetic variants on (**A**) indexed left atrial volumes in ml/m^2 and (**B**) left atrial passive and active phase emptying fractions from 35,658 cardiac MRIs in UK Biobank cohort. Boxes represent point estimates of effect per log odds higher genetically predicted risk of AF, with their size inversely proportional to variance and solid lines representing 95% confidence intervals (CI). Effect sizes were calculated using inverse variance weighted methods from summary GWAS data. For comparison to pathway scores see **Figure 3** in main paper.

Supplemental Figure S5. Associations between genetically predicted risk of AF via differing biological pathways and NT-proBNP levels in INTERVAL and IMPROVE cohorts

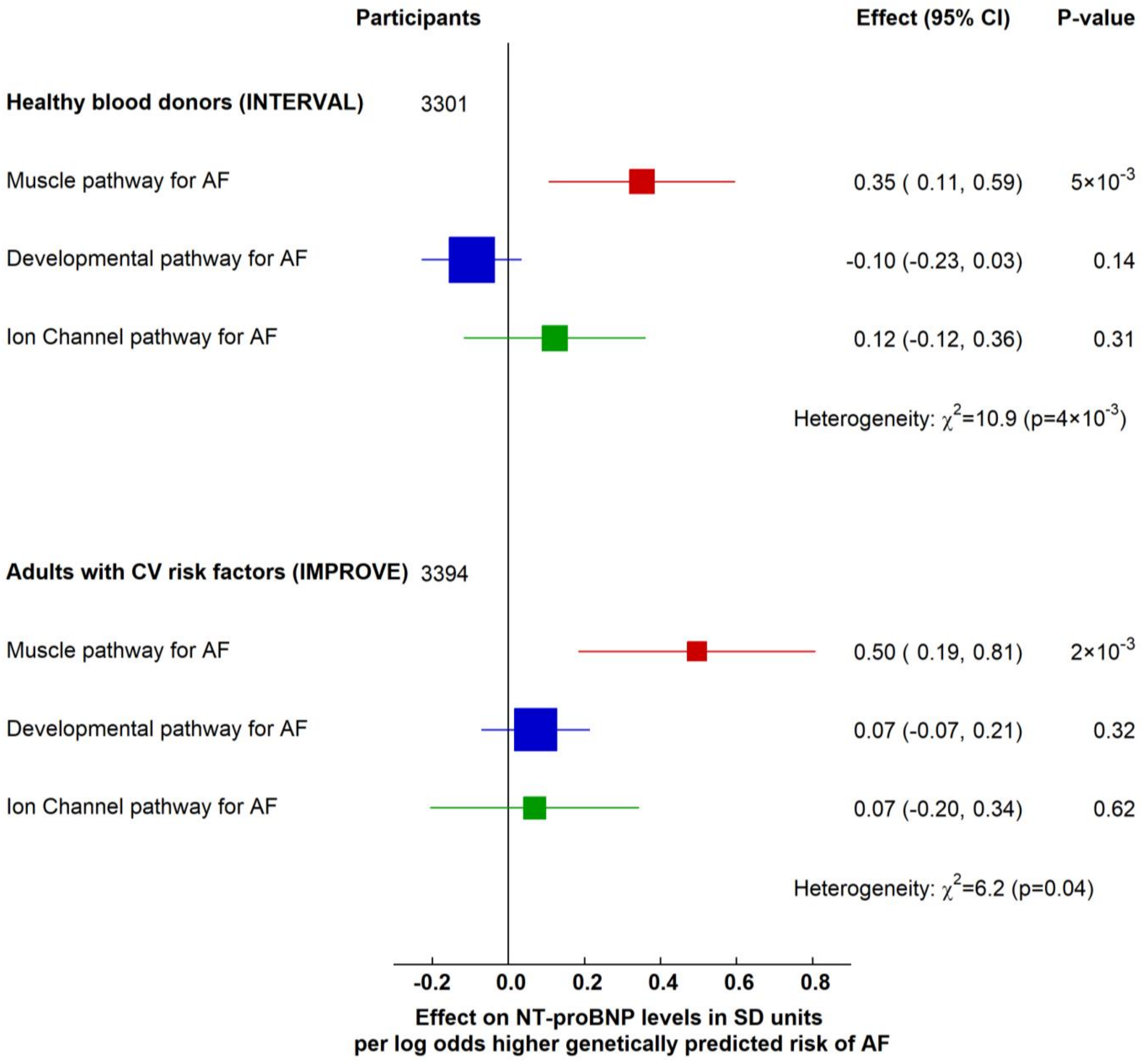

Effect of AF scores on N-terminal pro B-type natriuretic peptide (NT-proBNP) in standard deviation (SD) units in 3,301 healthy blood donors from the INTERVAL study, and 3,394 adults with cardiovascular (CV) risk factors from the IMPROVE study. Boxes represent point estimates of effect per log odds higher genetically predicted risk of AF, with their size inversely proportional to variance and solid lines representing 95% confidence intervals (CI). Effect sizes were calculated using inverse variance weighted methods using summary GWAS data, with heterogeneity tested using Cochran's Q statistic.

Supplemental Figure S6. Associations between comprehensive group of variants for AF risk and NT-proBNP levels

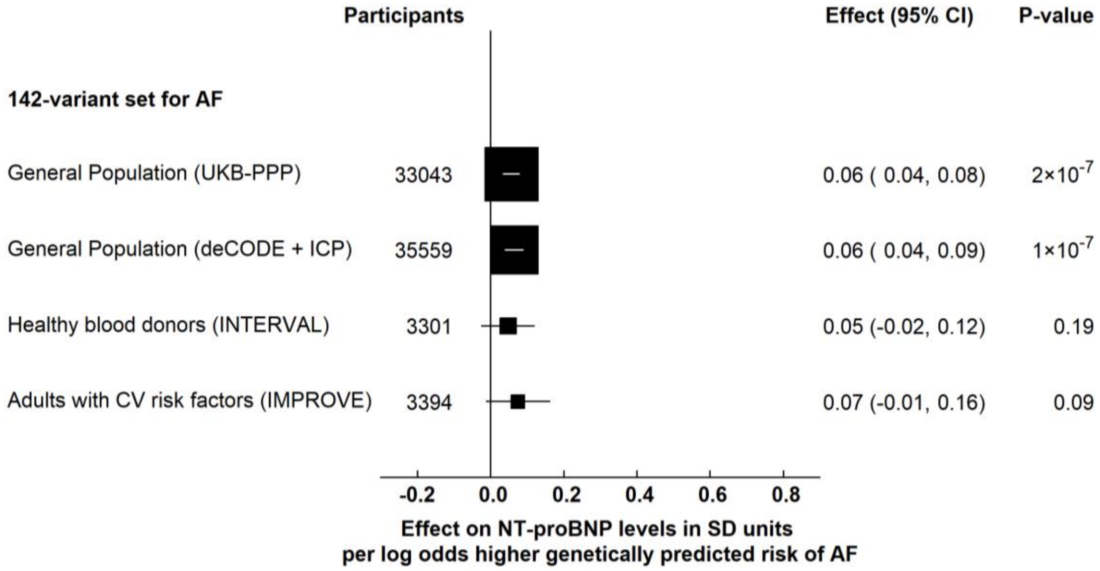

Effect of comprehensive atrial fibrillation (AF) risk predicting group of 142 independent genetic variants on N-terminal pro B-type natriuretic peptide (NT-proBNP) in standard deviation (SD) units in 33,043 mixed British individuals from the UK Biobank Pharma Proteomics Project (UKB-PPP), in 35,559 mixed Icelandic individuals from the deCODE and Icelandic cancer project (ICP), in 3,301 healthy blood donors from the INTERVAL study, and 3,394 adults with cardiovascular (CV) risk factors from the IMPROVE study. Boxes represent point estimates of effect per log odds higher genetically predicted risk of AF, with their size inversely proportional to variance and solid lines representing 95% confidence intervals (CI). Effect sizes were calculated using inverse variance weighted methods using summary GWAS data. For comparison to pathway scores see **Figure 4** in main paper and **Supplemental Figure S5**.

Supplemental Figure S7. Effects of genetically predicted AF biological pathways on high-sensitivity troponin I and T levels in different cohorts

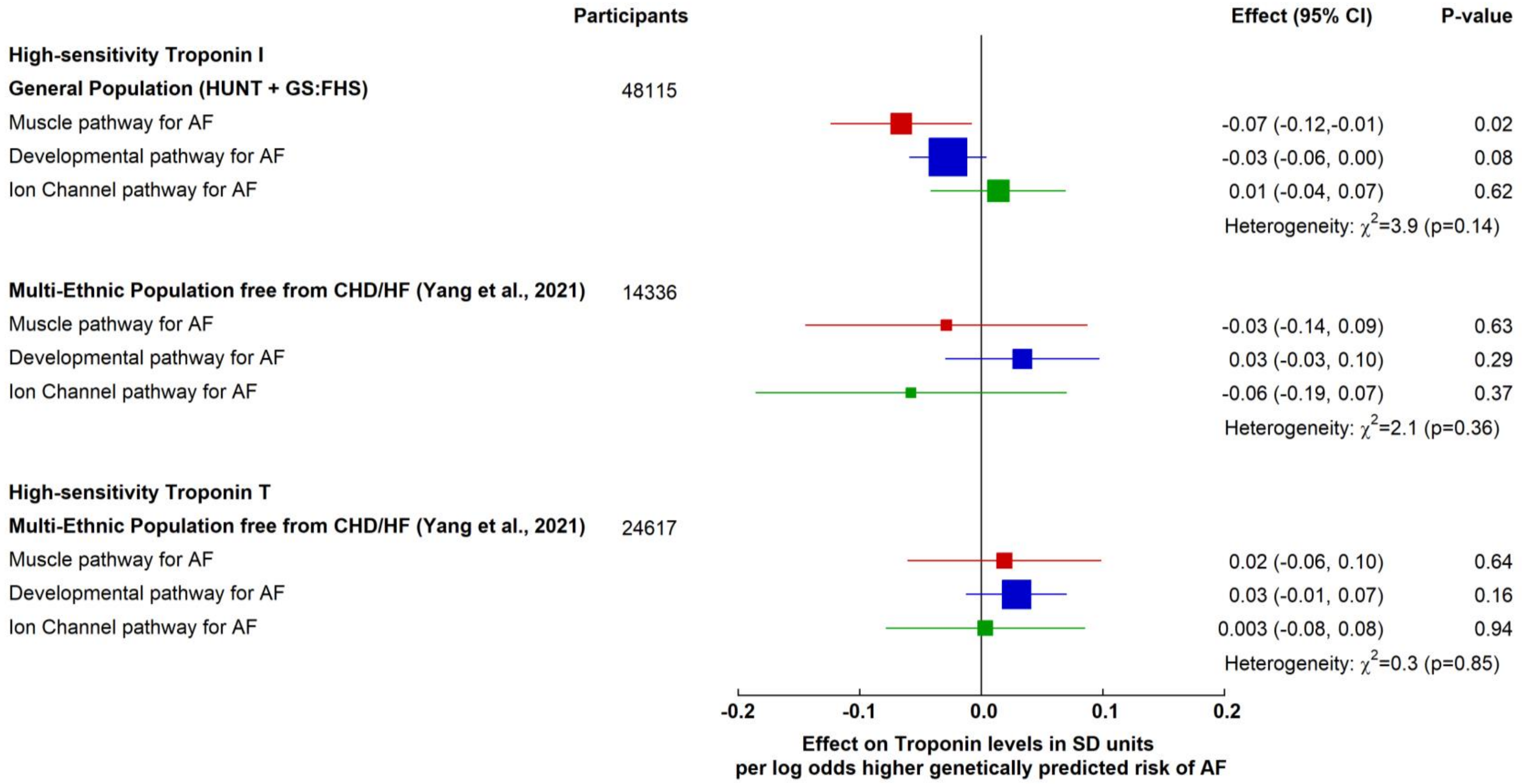

Effect of AF pathways on high-sensitivity troponin I and T levels in standard deviation (SD) units in 48,115 individuals from the HUNT and GS:FHS cohorts, and in a multi-ethnic population free from prevalent coronary heart disease (CHD) and heart failure (HF) as described in Yang *et al.*[35] Boxes represent point estimates of effect per log odds higher genetically predicted risk of AF, with their size inversely proportional to variance and solid lines representing 95% confidence intervals (CI). Effect sizes were calculated using inverse variance weighted methods using summary GWAS data with heterogeneity tested using Cochran's Q statistic.

Supplemental Figure S8. Associations between comprehensive group of variants for AF risk and high-sensitivity troponin I and T levels in different cohorts

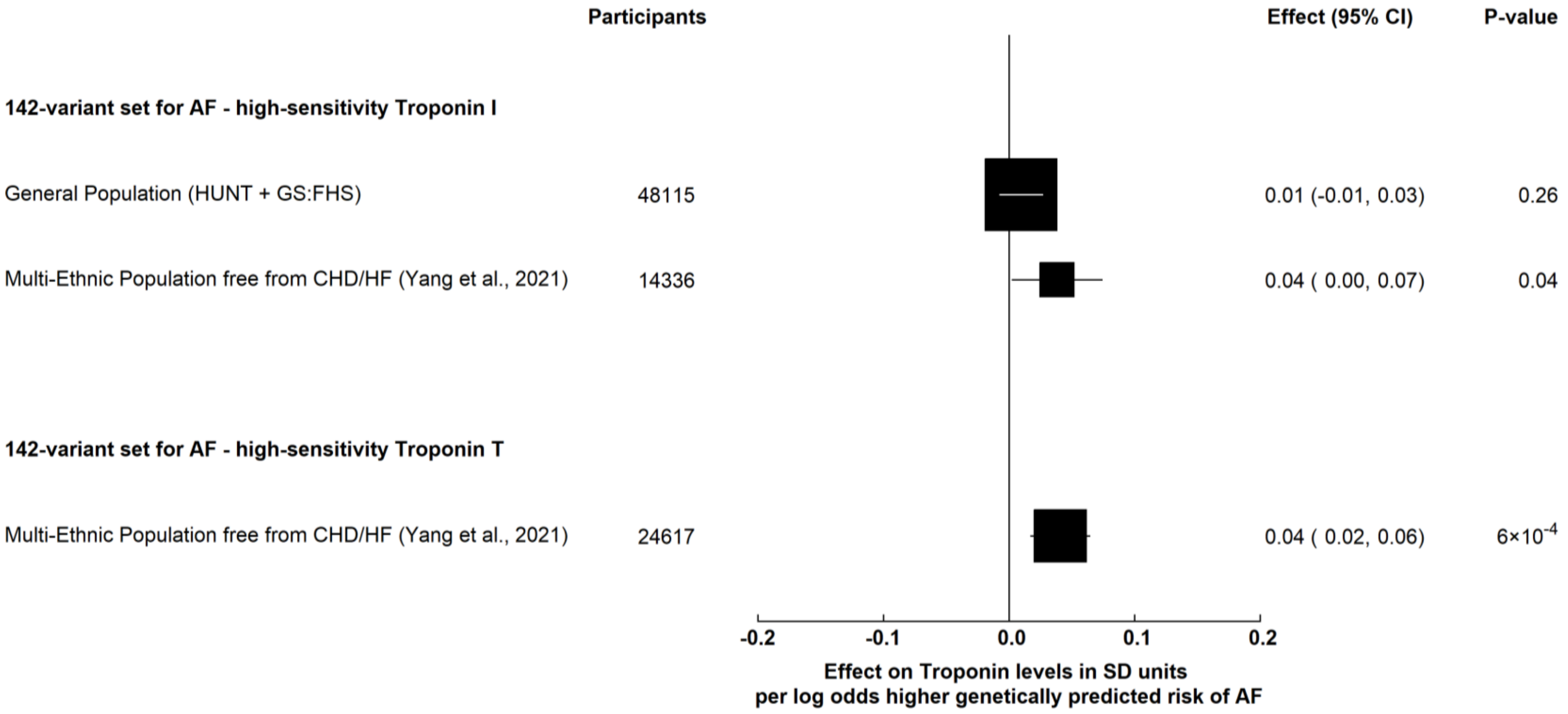

Effect of comprehensive atrial fibrillation (AF) risk predicting group of 142 independent genetic variants on high-sensitivity troponin I and T levels in standard deviation (SD) units in 48,115 individuals from the HUNT and GS:FHS cohorts, and in a multi-ethnic population free from prevalent coronary heart disease (CHD) and heart failure (HF) as described in Yang *et al*., Circ Genom Precis Med 2021. Boxes represent point estimates of effect per log odds higher genetically predicted risk of AF, with their size inversely proportional to variance and solid lines representing 95% confidence intervals (CI). Effect sizes were calculated using inverse variance weighted methods using summary GWAS data. For comparison to pathway scores see **Supplemental Figure S7**.

Supplemental Figure S9. Associations between genetically predicted AF biological pathways and small-vessel ischaemic stroke

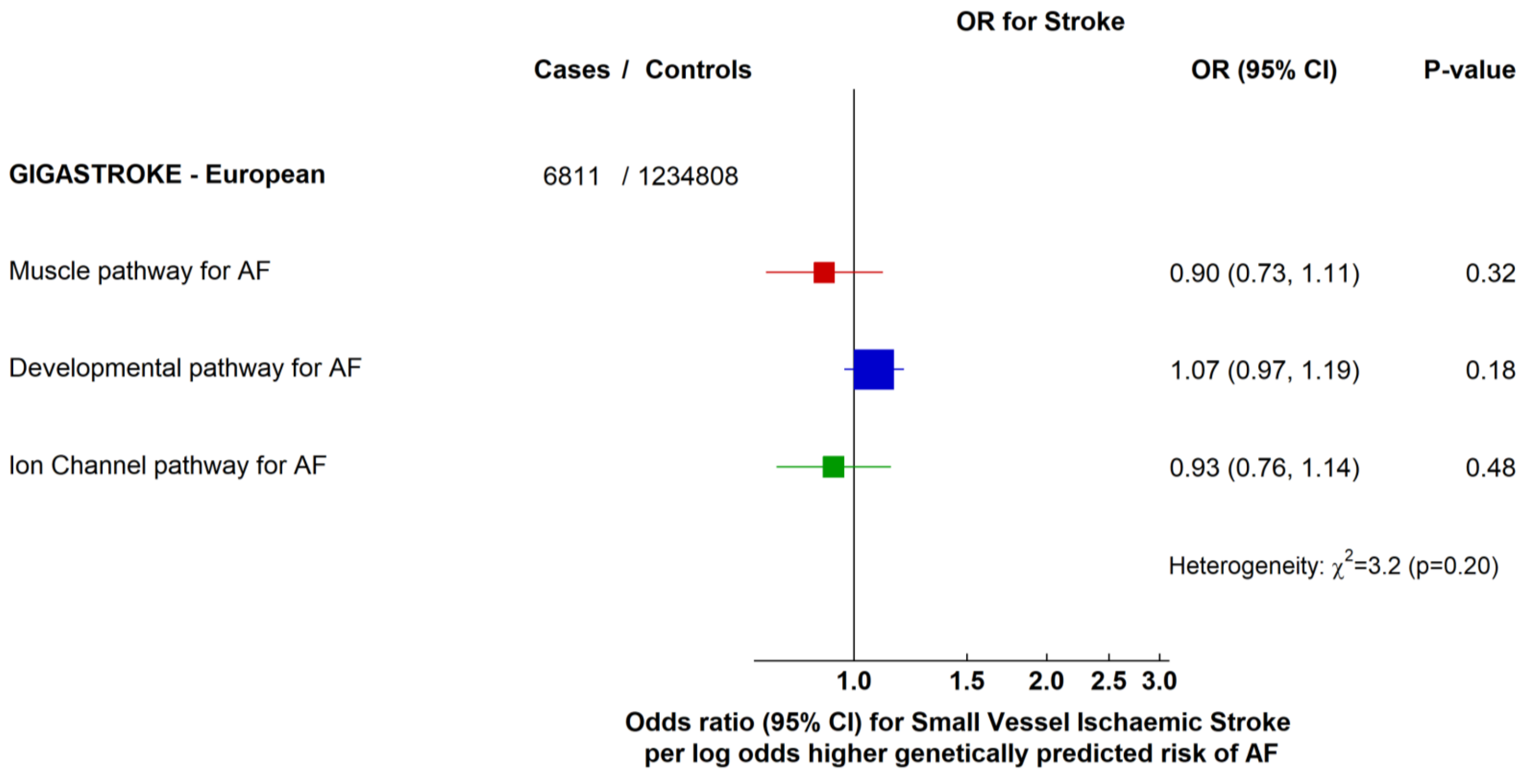

Odds ratio (OR) for developing small-vessel ischaemic stroke in the GIGASTROKE dataset. Boxes represent point estimates of effect per log odds higher genetically predicted risk of atrial fibrillation (AF), with their size inversely proportional to variance and solid lines representing 95% confidence intervals (CI). ORs were calculated using inverse variance weighted methods from summary GWAS data, and heterogeneity tested using Cochran's Q statistic.

Supplemental Figure S10. Associations between comprehensive group of variants for AF risk and different types of ischaemic stroke in GIGASTROKE cohort

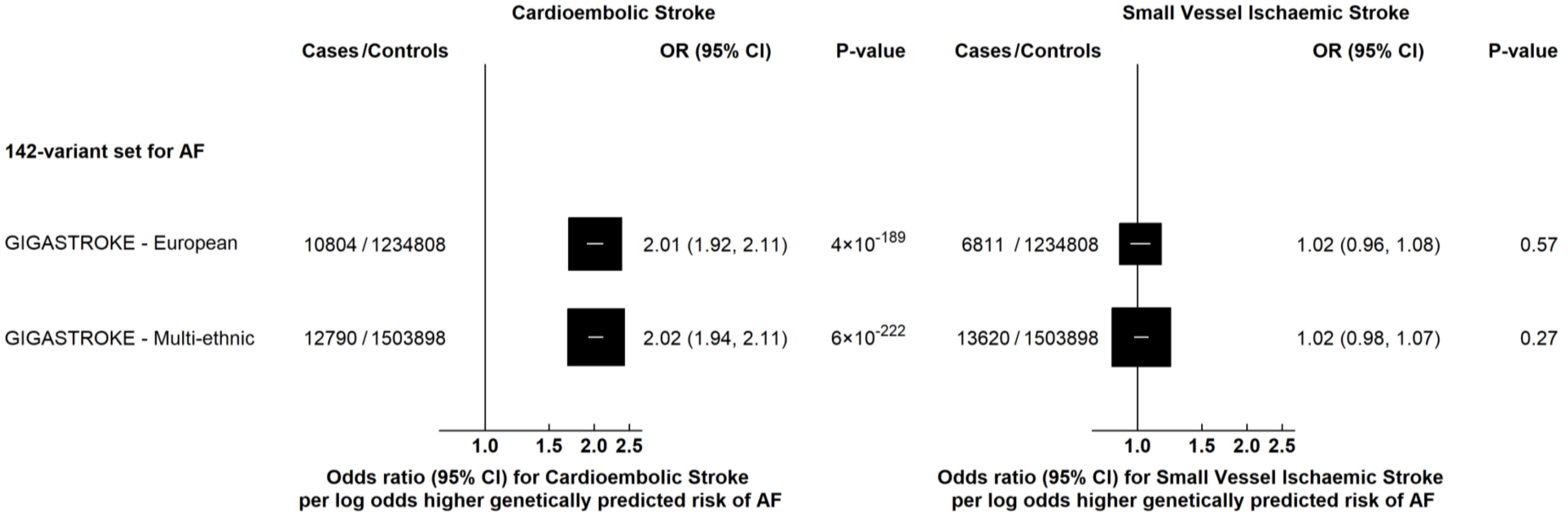

Odds ratio (OR) for developing types of ischaemic stroke in the GIGASTROKE dataset using a comprehensive atrial fibrillation (AF) risk predicting group of 142 independent genetic variants. Boxes represent point estimates of effect per log odds higher genetically predicted risk of AF, with their size inversely proportional to variance and solid lines representing 95% confidence intervals (CI). ORs were calculated using inverse variance weighted methods from summary GWAS data. For comparison to pathway scores see **Figure 5** in main paper.

Supplemental Figure S11. Funnel plot for SNPs in muscle pathway for AF

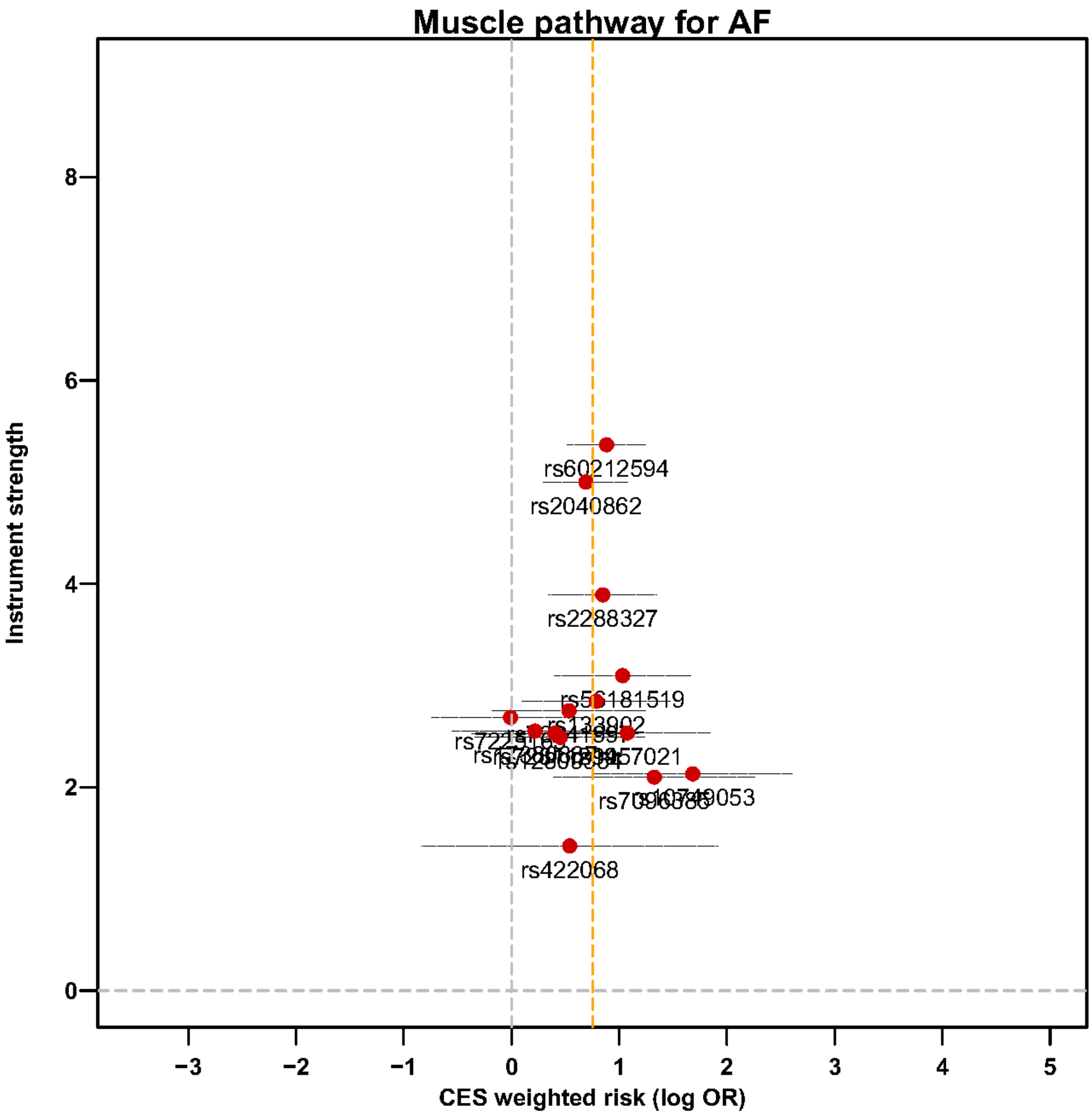

Funnel plot showing individual single nucleotide polymorphism (SNP) effects on cardio-embolic stroke (CES) expressed as log odds ratio (OR) estimate from GIGASTROKE data vs. strength of SNP in genetic score for atrial fibrillation via muscle pathway (effect on AF / standard error of estimate of effect on CES). Orange dotted line is the overall effect estimate for the score.

Supplemental Figure S12. Funnel plot for SNPs in developmental pathway for AF

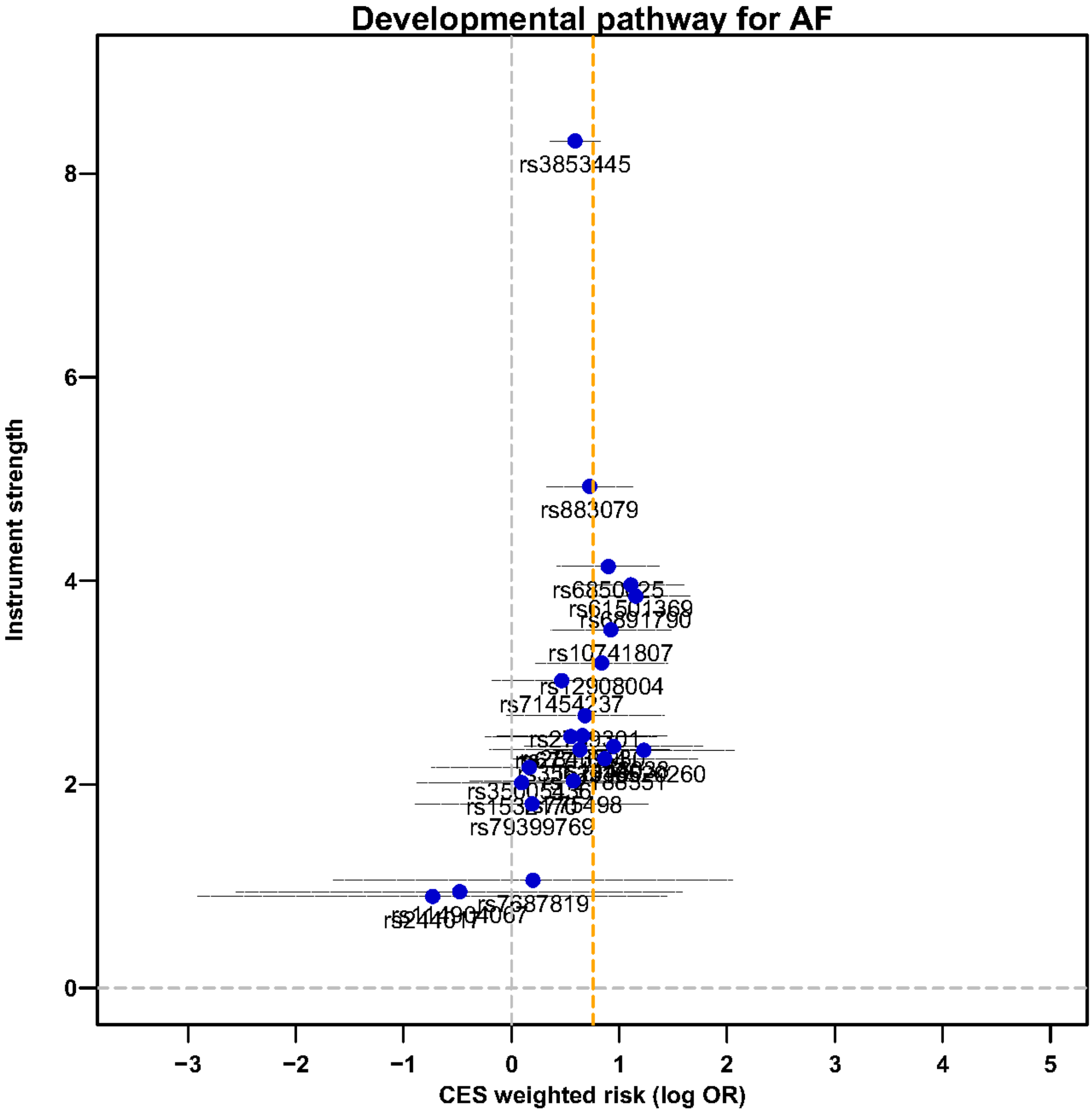

Funnel plot showing individual single nucleotide polymorphism (SNP) effects on cardio-embolic stroke (CES) expressed as log odds ratio (OR) estimate from GIGASTROKE data vs. strength of SNP in genetic score for atrial fibrillation via developmental pathway (effect on AF / standard error of estimate of effect on CES). Orange dotted line is the overall effect estimate for the score.

Supplemental Figure S13. Funnel plot for SNPs in ion channel pathway for AF

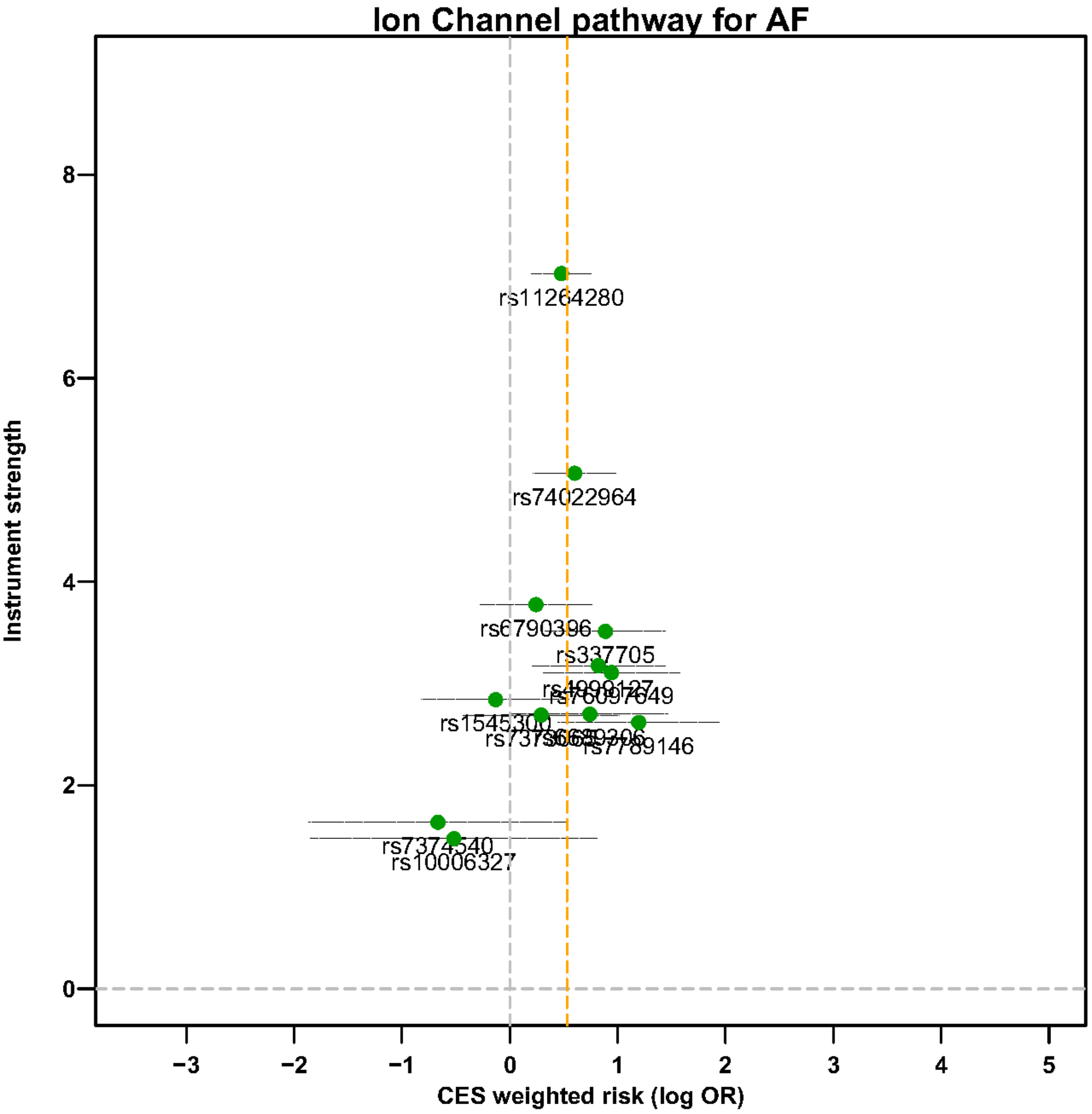

Funnel plot showing individual single nucleotide polymorphism (SNP) effects on cardio-embolic stroke (CES) expressed as log odds ratio (OR) estimate from GIGASTROKE data vs. strength of SNP in genetic score for atrial fibrillation via ion channel pathway (effect on AF / standard error of estimate of effect on CES). Orange dotted line is the overall effect estimate for the score.

Supplemental Figure S14. Leave one out analyses for SNPs in muscle pathway for AF and cardioembolic stroke

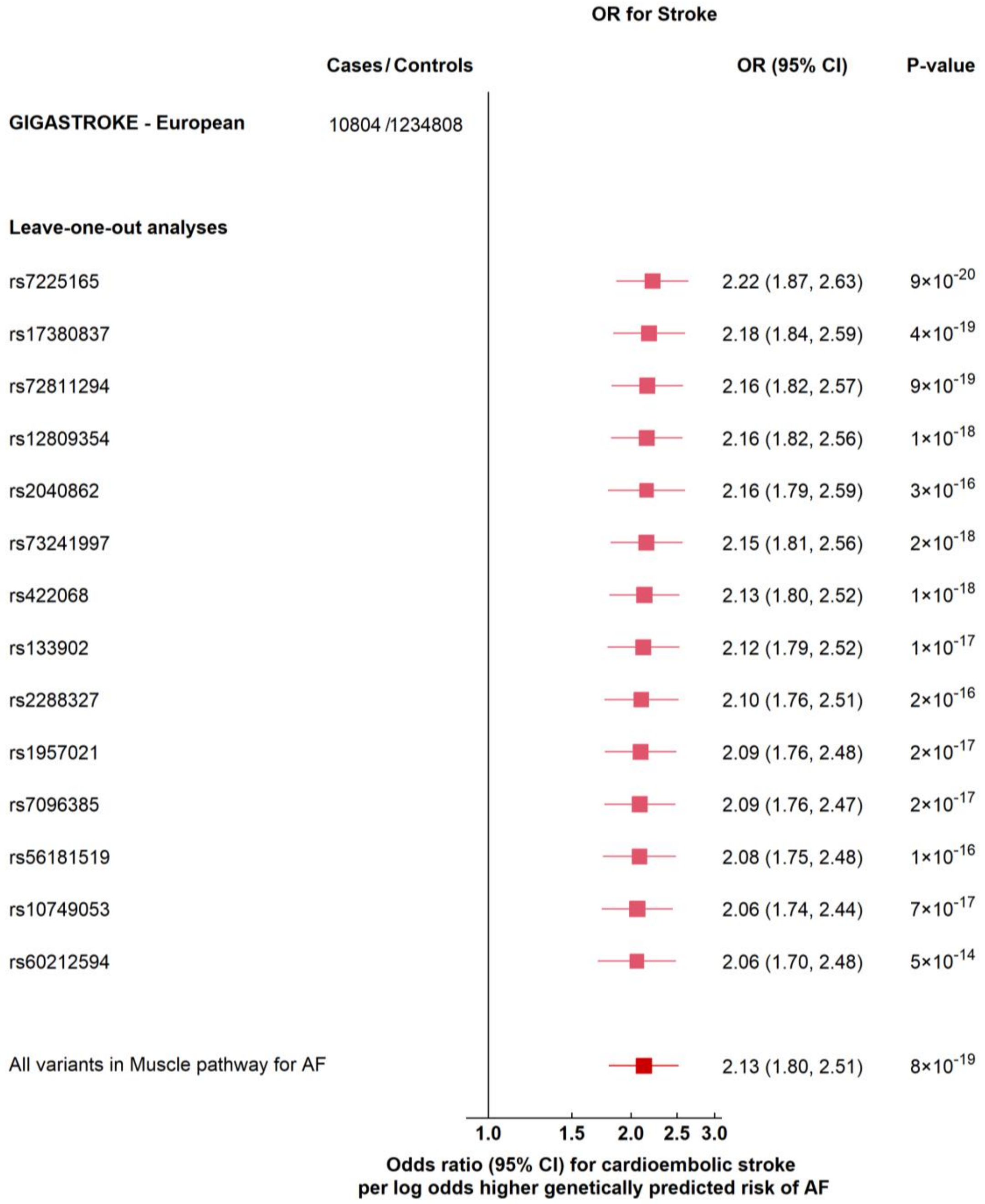

Odds ratio (OR) for developing cardioembolic ischaemic stroke in the GIGASTROKE dataset via the Muscle pathway for atrial fibrillation (AF), with individual single nucleotide polymorphisms (SNPs) excluded in leave-one-out analyses. Data available in GIGASTROKE for 14/15 SNPs (see **Supplemental Table S2**). Boxes represent point estimates of effect per log-odds higher genetically predicted risk of AF with their size inversely proportional to variance and solid lines representing 95% confidence intervals (CI). ORs were calculated using inverse variance weighted methods from summary GWAS data.

Supplemental Figure S15. Leave one out analyses for SNPs in developmental pathway for AF and cardioembolic stroke

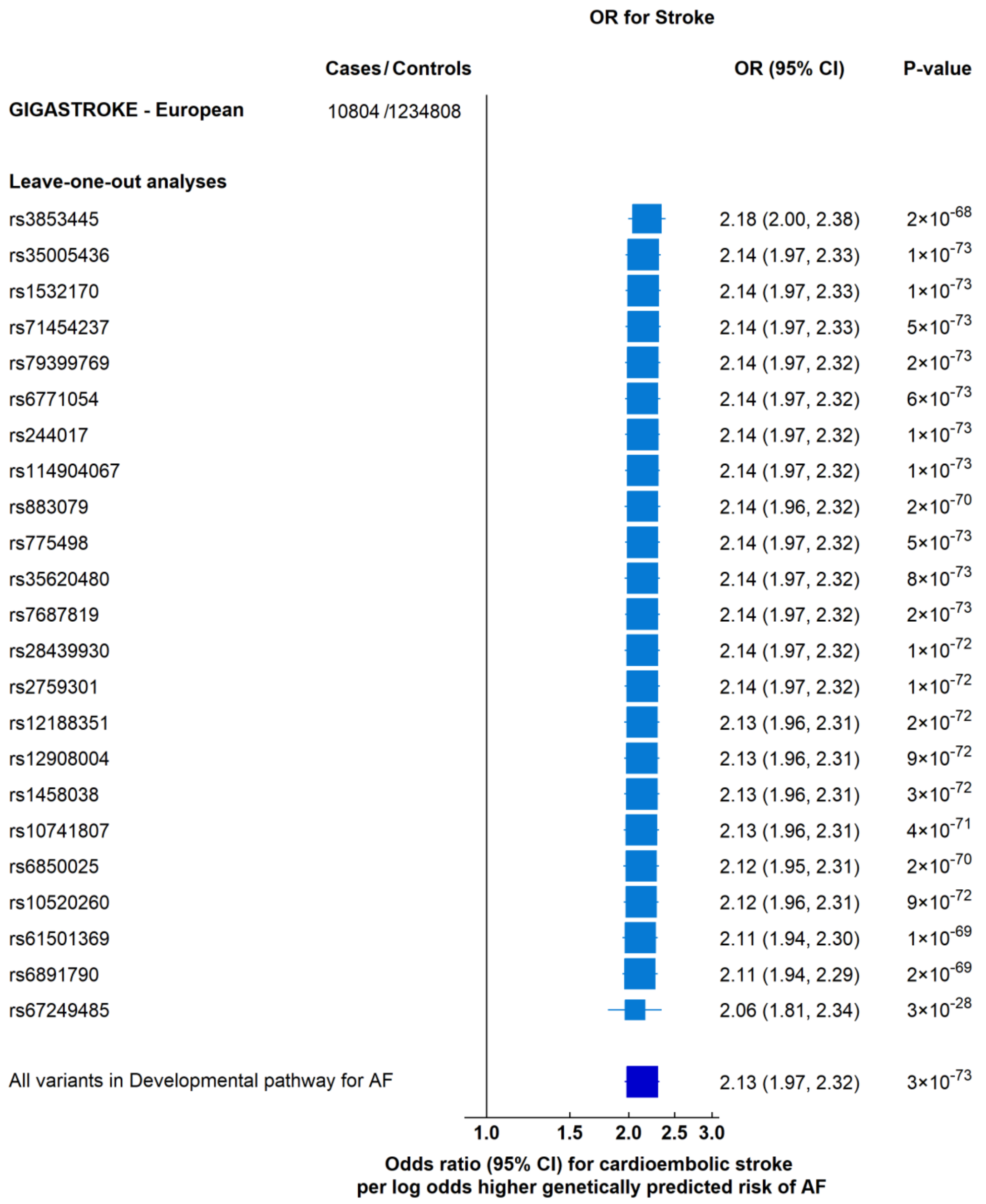

Odds ratio (OR) for developing cardioembolic ischaemic stroke in the GIGASTROKE dataset via the Developmental pathway for atrial fibrillation (AF), with individual single nucleotide polymorphisms (SNPs) excluded in leave-one-out analyses. Data available in GIGASTROKE for 23/25 SNPs (see **Supplemental Table S3**). Boxes represent point estimates of effect per log-odds higher genetically predicted risk of AF, with their size inversely proportional to variance and solid lines representing 95% confidence intervals (CI). ORs were calculated using inverse variance weighted methods from summary GWAS data.

Supplemental Figure S16. Leave one out analyses for SNPs in ion channel pathway for AF and cardioembolic stroke

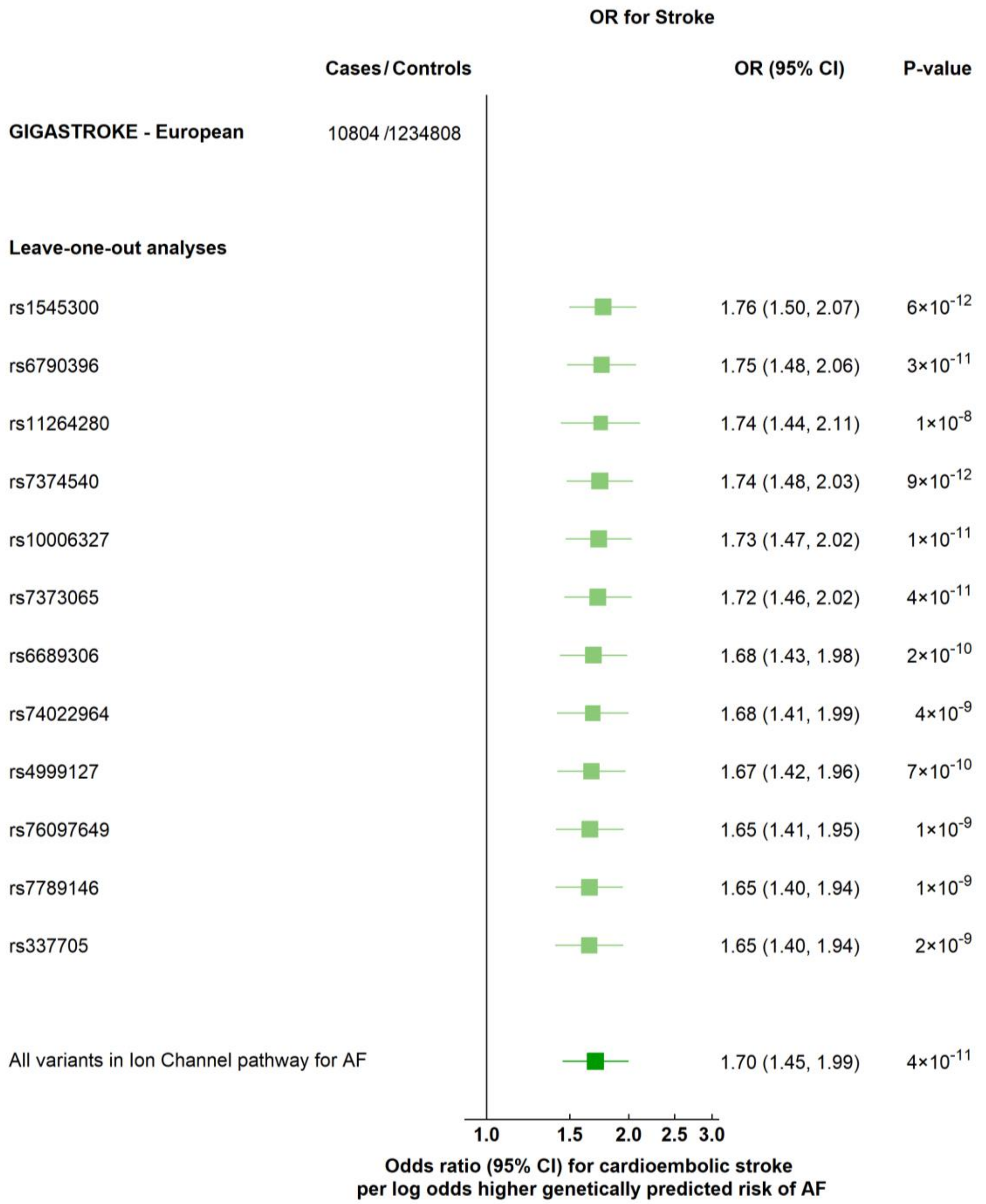

Odds ratio (OR) for developing cardioembolic ischaemic stroke in the GIGASTROKE dataset via the Developmental pathway for atrial fibrillation (AF), with individual single nucleotide polymorphisms (SNPs) excluded in leave-one-out analyses. Data available in GIGASTROKE for all 12 SNPs. Boxes represent point estimates of effect per log-odds higher genetically predicted risk of AF, with their size inversely proportional to variance and solid lines representing 95% confidence intervals (CI). ORs were calculated using inverse variance weighted methods from summary GWAS data.
